# Deep learning-enhanced nuclear medicine SPECT imaging applied to cardiac studies

**DOI:** 10.1186/s40658-022-00522-7

**Published:** 2023-01-27

**Authors:** Ioannis D. Apostolopoulos, Nikolaos I. Papandrianos, Anna Feleki, Serafeim Moustakidis, Elpiniki I. Papageorgiou

**Affiliations:** 1 Department of Medical Physics, School of Medicine, University of Patras, 26504 Patras, Greece; 2 Department of Energy Systems, University of Thessaly, Gaiopolis Campus, 41500 Larisa, Greece; 3 AIDEAS OÜ, 10117 Tallinn, Estonia

**Keywords:** Deep learning, Cardiovascular diseases, Nuclear medicine, SPECT, Artificial intelligence

## Abstract

Deep learning (DL) has a growing popularity and is a well-established method of artificial intelligence for data processing, especially for images and videos. Its applications in nuclear medicine are broad and include, among others, disease classification, image reconstruction, and image de-noising. Positron emission tomography (PET) and single-photon emission computerized tomography (SPECT) are major image acquisition technologies in nuclear medicine. Though several studies have been conducted to apply DL in many nuclear medicine domains, such as cancer detection and classification, few studies have employed such methods for cardiovascular disease applications. The present paper reviews recent DL approaches focused on cardiac SPECT imaging. Extensive research identified fifty-five related studies, which are discussed. The review distinguishes between major application domains, including cardiovascular disease diagnosis, SPECT attenuation correction, image denoising, full-count image estimation, and image reconstruction. In addition, major findings and dominant techniques employed for the mentioned task are revealed. Current limitations of DL approaches and future research directions are discussed.

## Introduction

Nuclear Medicine is a modern medicine speciality characterized by the use of small amounts of radioactive substances or radiodiagnostic reagents for diagnostic and therapeutic purposes [1]. The most common nuclear medicine application is the scintigraphy diagnostic test. Several imaging modalities belong to the scope of medical imaging in nuclear medicine, such as positron emission tomography (PET) and single-photon emission computerized tomography (SPECT). Radioisotope imaging of myocardial perfusion is a set of imaging techniques for assessing perfusion and myocardial function, both at rest and during physical or pharmacological exercise, to diagnose and manage patients with known or potential coronary heart disease. It is achieved by administering a radioactive marker (radioisotope), usually intravenously, and using a special camera system (γ-camera), usually SPECT, or PET, which detects the emission photons [1]. Images of myocardial perfusion are taken at rest and after fatigue/loading, which using specific software, are reconstructed into given images.

Recent advances in artificial intelligence (AI) enable high-level image processing and have gained research attention [2]. The exponential growth of data that has taken place in recent decades and the rapid development of the computing power of modern computer systems have been the determining factors for developing new methods and techniques in image processing and analysis. From the traditional and well-established AI methods, such as artificial neural networks (ANNs) [3] and support vector machines (SVMs) [4], to the more sophisticated and deep networks, such as convolutional neural networks (CNNs) [5] and generative adversarial networks (GANs) [6], several applications concern the field of nuclear medicine, especially in myocardial perfusion imaging (MPI).

Cardiovascular diseases are the leading cause of death in the EU, accounting for 45% of these deaths in females and 39% in males [7]. They cover many medical problems affecting the circulatory system (the heart and blood vessels). Some of the most common diseases that affect the circulatory system include ischaemic heart disease (heart attacks) and cerebrovascular diseases (strokes) [7]. Heart attacks and strokes are usually acute events mainly caused by a blockage that prevents blood from flowing to the heart or brain. The most common reason for this is a build-up of fatty deposits on the inner walls of the blood vessels that supply the heart or brain. Strokes can be caused by bleeding from a blood vessel in the brain or by blood clots.

A literature review has assigned the detection and classification of various diseases among the top applications of AI in MPI imaging. SPECT image denoising, artefact removal, and low-count SPECT reconstruction are important application domains. In the present review paper, we focus on advanced deep learning (DL) [8] methods in cardiac imaging and, more specifically, the myocardial perfusion imaging delivered by the SPECT scanners. Thorough research identified fifty-five studies dealing with this topic. Αmong the purposes of the paper is to provide a review for readers new to the field of AI.

The reviewed papers fall under two major categories: Cardiovascular disease diagnosis via image and clinical data classification methods and image quality improvement. For cardiovascular disease diagnosis, studies employing explainability methods are highlighted and analytically discussed. Per-patient and per-vessel outcomes are also assessed quantitatively and qualitatively. Image quality improvement includes several domains of SPECT imaging. The main objective of image quality improvement is to generate an improved SPECT image or complete scan liberated from artefacts, noise, and attenuation. In addition, estimating the full-count SPECT scan from its low-count counterpart is a notable image improvement that does not intend to remove the noise solely but also to estimate the radiation outcome of a hypothetical full-dose input. Studies relating to the above domains are discussed based on their objectives and results. The review study classifies the research papers according to their DL methods, their validation procedure in terms of data size and evaluation method, and their quantitative results. In the Discussion section, the study presents the most significant results and highlights major limitations that need future examination.

The present paper is organized into the following sections: "Machine learning in a nutshell: definitions and terminology" section presents a brief description of the dominant machine learning (ML) and DL methods found in the literature. In "Materials and methods" section, the review methodology, inclusion and exclusion criteria, as well as the literature sources are described. The major findings of the studies that this review covers are extensively presented in "Results" section. Finally, discussion and concluding remarks are provided in "Concluding remarks" section.

## Machine learning in a nutshell: definitions and terminology

To aid readers' comprehension and for completeness, this section rapidly summarizes the pertinent terminology and definitions for the ML and DL algorithms utilized in the research included in this review. ML is a subfield of artificial intelligence that focuses on creating algorithms that automatically learn to generate correct predictions via experience (data) rather than hard-coded instructions.

Supervised ML systems work in two phases: learning (training) and testing. A feature extraction/selection step (sometimes referred to as feature engineering) is applied first in a standard ML pipeline to extract or identify the most useful features [9]. The retrieved characteristics are then fitted to an ML model, and the best model parameters are determined. During the testing step, the trained model is supplied with previously unseen samples (either as pictures or features derived from images) for classification. Unlike traditional programming, where rules are manually created, supervised ML algorithms build rules from data.

DL [8] is a subsection of ML that shifts the focus from visual handcrafted feature extraction to the underlying learning mechanism. CNNs are a family of DL techniques often employed in computer vision and pattern recognition applications. They are actually a type of neural network constructed of four types of layers: (1) input layer, (2) convolution layer, (3) pooling layer, and (4) fully connected layer. The convolution layers use filters that execute convolution operations on the input as it is scanned in terms of dimensions. Pooling is a downsampling procedure generally used in conjunction with a convolution layer. Fully connected layers operate with flattened inputs that connect all neurons in the following layer. They are often seen towards the end of CNN designs to maximize class scores. Handcrafted CNNs are usually lightweight, problem-specific designed networks that are trained from scratch using the training data of the domain they are designed to operate in.

Transfer learning is a technique in which a network trained on a large dataset is partly reused to address a new problem. The key idea behind transfer learning is that generic characteristics learned on a large dataset may be applied to various domain problems with fewer data. Among the most widely used networks are pre-trained networks, such as DenseNet [10], AlexNet [11], and VGG [12]. Because the pre-trained network is an arbitrary feature extractor, the input image is passed through many layers before arriving at a pre-specified layer, the outputs of which are the final extracted features.

Numerous CNN-based algorithms have been recently developed, enabling us to attain incredible accuracy on various problems, even outperforming human performance. 3D CNNs [13] are essentially the three-dimensional extension of two-dimensional CNNs. They accept as input a three-dimensional volume or a series of two-dimensional frames (e.g., slices in an MRI scan). Then, kernels traverse three dimensions of data, yielding three-dimensional activation maps. In general, they build sophisticated representations of volumetric data. Graph CNNs (GCNN) are a class of neural networks that function natively on graph-structured data [14]. GCNNs may generate more informative predictions about entities in these interactions by extracting and exploiting information from the underlying graph, as opposed to models that analyze individual entities in isolation. GANs [6] are a powerful type of neural network that is used for unsupervised learning. It consists primarily of a system of two competing neural network models that can assess, capture, and replicate the variances within a dataset. Finally, U-NET [15] is one of the most frequently utilized techniques in semantic segmentation nowadays. U-NET is an encoder-decoder network architecture that has the form of a U and is composed of four encoder blocks and four decoder blocks that are linked together by a bridge. Following the aforementioned CNN-based model's cutting-edge potential, many variants have been proposed based on variations in convolution and pooling operations, skip connections, and the arrangement of the components in each layer to address the challenges associated with various applications.

## Materials and methods

To conduct the systematic literature review, a three-step process of plan, conduct, and report has been observed. In the planning phase, research questions were defined, and the review protocol was established, specifying the publication sources, search terms, and selection criteria. In the second step, the literature was collected following the review protocol. The selected literature was analyzed, extracting and synthesizing the required data to answer the questions. Finally, the review results were documented, addressing the research questions and the objectives of the systematic literature review.

### Research questions

The main objective of this review was to determine how DL has been applied for CAD in nuclear medicine using SPECT scans. Furthermore, to look into the applications and how the CAD methods have been implemented using the deep networks. Thus, providing the knowledge of the current practices to build upon that for further improvement in the area. Therefore, the following three research questions (RQs) have been framed:

1. What AI/DL learning algorithms have been applied?
2. What kind of architecture of the deep network has been employed?
3. What was the nature of the network training and testing data?

Α focused approach has been followed while scanning the literature. Each article has been reviewed to answer the above questions. The gathered data has been reported in a comprehensive way to have a complete picture.

### Review protocol

#### Search sources and terms

**Fig. 1:**
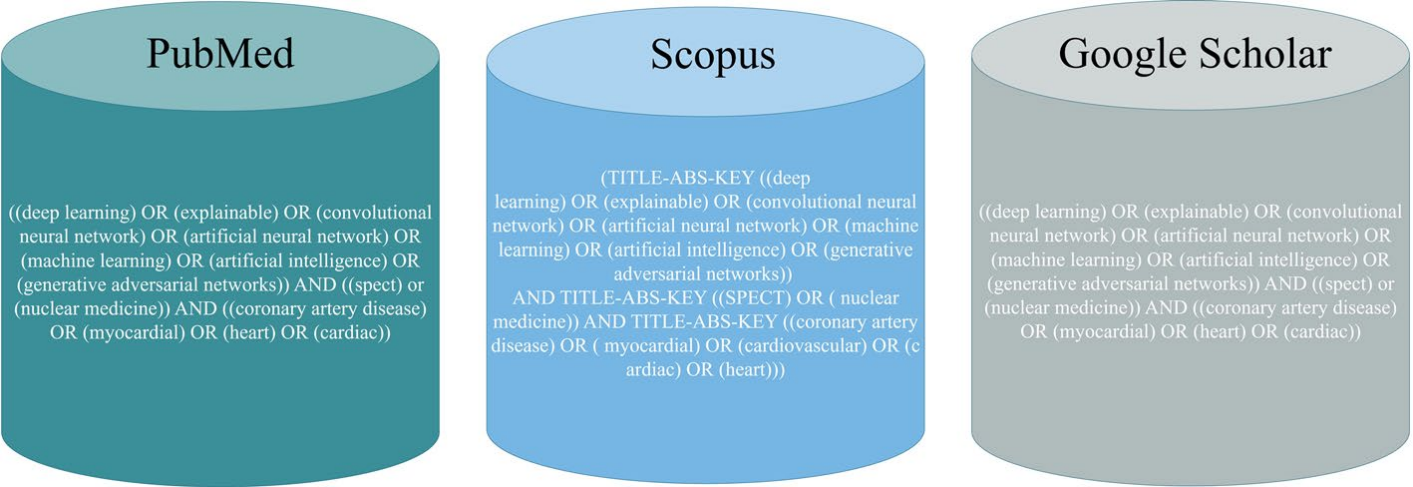
Utilized keywords in scientific search databases

Three popular scientific databases, Pubmed, Scopus, and Google Scholar, were selected to extract the data. The investigated topic combines three main search terms: ‘Deep Learning’, ‘SPECT MPI’, and ‘Cardiovascular’. Each of the terms can be searched by multiple alternative words. The most relevant and commonly used applicable terms were selected and combined by the ‘OR’ operator. For example, to represent ‘deep learning’, three search terms were identified: ' deep learning’, ‘deep network’, and ‘deep architecture’. The other term for ‘SPECT MPI’ and ‘nuclear medicine’ was represented by their only main term. Individual search strings were concatenated by the ‘AND’ operator to form a search query. The wild card ‘∗’ has been added to include all verb forms of the key terms. Full-text search has been employed to capture the maximum relevant literature. Complete search queries for each of the databases are shown in Fig. 1.

Search String in PubMed: ((deep learning) OR (explainable) OR (convolutional neural network) OR (artificial neural network) OR (machine learning) OR (artificial intelligence) OR (generative adversarial networks)) AND ((spect) or (nuclear medicine)) AND ((coronary artery disease) OR (myocardial) OR (heart) OR (cardiac)).

Search string in Scopus: (TITLE-ABS-KEY (("deep learning ") OR ("explainable") OR ("convolutional neural network") OR ("artificial neural network") OR ("machine learning") OR ("artificial intelligence") OR ("generative adversarial networks")) AND TITLE-ABS-KEY (("SPECT") OR ("nuclear medicine")) AND TITLE-ABS-KEY (("coronary artery disease") OR ("myocardial") OR ("cardiovascular") OR ("cardiac") OR ("heart"))).

#### Inclusion and exclusion

This study is limited to DL applications for cardiovascular diagnosis in nuclear medicine. All primary studies published in English employing a DL algorithm for cardiovascular classification, identification, attenuation correction, or any other CAD diagnosis task were included. No limits on the subject areas and time frame were imposed for a broad search spectrum. However, since DL is an emerging field, the literature returned in response to the search queries spanned over recent years, starting from 2017 onwards. The time period of the selected articles extends over 4 years, from 2017 to June 2022. The chosen literature included journal articles, conference proceedings, and book sections on the explored topic.

- Articles published after 2017.
- CAD detection was conducted via AI algorithms, with traditional ML and DL approaches included.
- Articles utilizing SPECT dataset for CAD detection.
- Original research articles (Journals, conferences).
- Papers not investigating PET or PET/CT cases for CAD.

This literature review includes studies on cardiovascular diagnosis represented by SPECT MPI images in nuclear medicine. The publications on other forms of images like PET or CT images or any other format were not included. The exclusion criteria are the following:

- Articles not related to AI/ML/CAD.
- Articles published before 2017.
- Non-original research articles (reviews, editorials, meta-analysis).
- Papers describing framework, platforms, software.
- Papers investigating PET or PET/CT cases for CAD.

### Literature collection

**Fig. 2:**
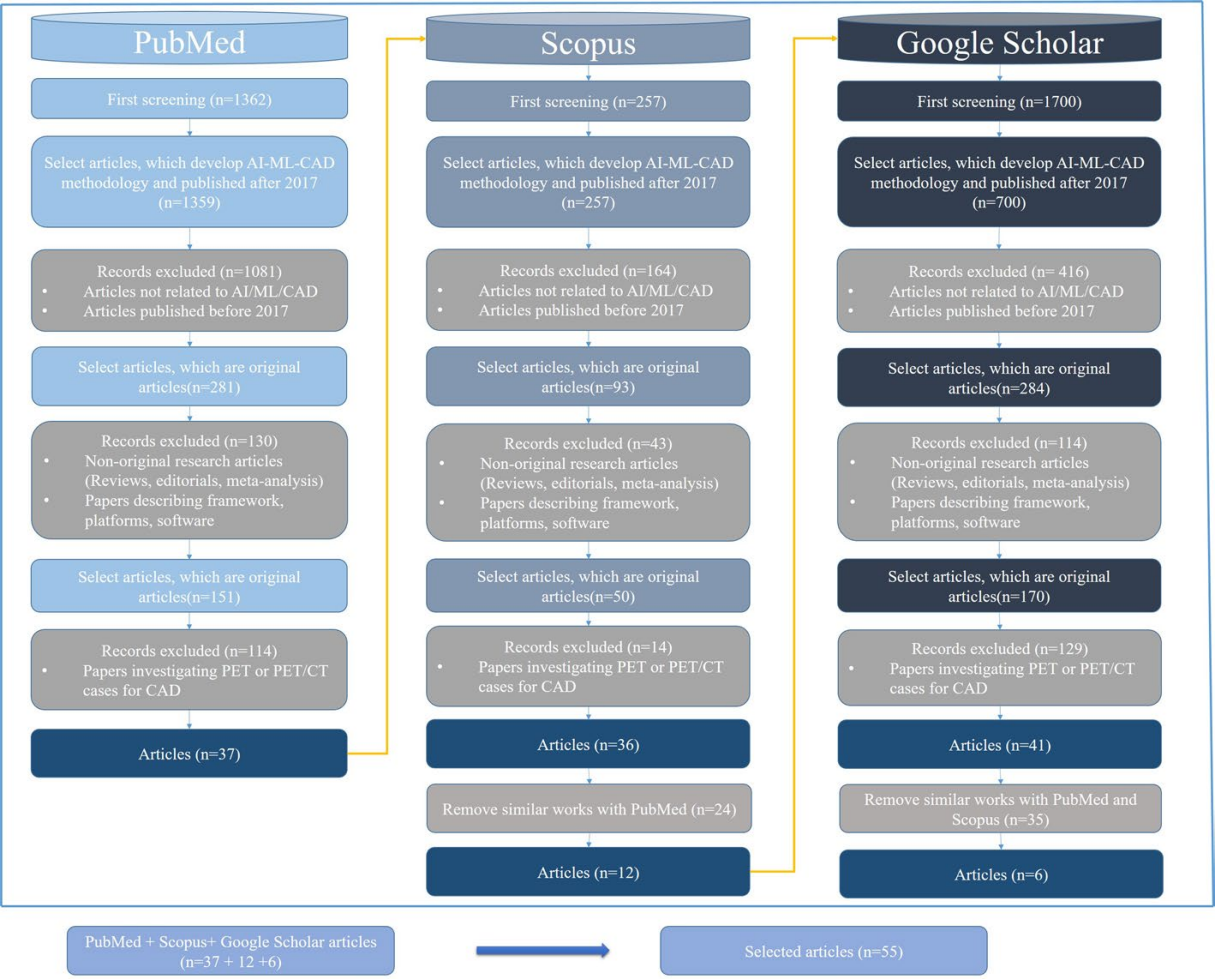
Literature search and qualification process

The literature search was performed by supplying the search strings for each database, as shown in Fig. 1. Many publications were returned in response to these search queries. The search results from each database were assessed according to the predefined inclusion/exclusion criteria. In the initial screening, the review articles and non-English publications were excluded. Each article was evaluated based on its title, abstract and a quick review of the text to decide its selection or rejection. This filtration reduced the number of articles to 114. After removing the duplicate articles, 71 publications were included in the full-text assessment, and finally, 55 studies were selected to be part of this literature review. Preferred Reporting Items have shown the data selection process for the Systematic Review and Meta-analysis (PRISMA) framework [16]. The complete process is visualized in Fig. 2.

### Types of outcomes measures

Multiple evaluation criteria and metrics have been reported. The model’s accuracy and the Area Under Curve (AUC) score obtained from the Receiver Operating Characteristic (ROC) curve are the most considered for evaluating the classification performance of the developed DL algorithms. The sensitivity score (sensitivity = TP/(TP + FN) and the specificity score (TN/(TN + FP)) of the developed models are also reported, where TP, FN, FP and TN denote true positives, false negatives, false positives and true negatives, respectively.

For the evaluation of the image quality improvement, several criteria are considered and discussed as follows:

- Peak signal-to-noise ratio (PSNR) expresses the ratio between the maximum possible value and the power of distorted noise. It is used to measure the quality of a generated or compressed image.
- Structural similarity index measure (SSIM) measures the similarity between two images.
- Mean error (ME): it expresses the pixel-to-pixel or voxel-to-voxel difference between a reference image and a DL-generated one
- Root-mean-squared error (RSME): it expresses the pixel-to-pixel or voxel-to-voxel square difference between a reference image and a DL-generated one
- Normalized mean error (nME): it expresses the pixel-to-pixel or voxel-to-voxel normalized difference between a reference image and a DL-generated one
- Normalized root-mean-squared error (nRSME): it expresses the pixel-to-pixel or voxel-to-voxel normalized square difference between a reference image and a DL-generated one
- Absolute relative error (ARE) [17]: it expresses the pixel-to-pixel or voxel-to-voxel absolute normalized difference between a reference image and a DL-generated one
- Noise level measured as the normalized standard deviation (NSD): It measures the normalized standard deviation of the grey values of the image

## Results

**Fig. 3:**
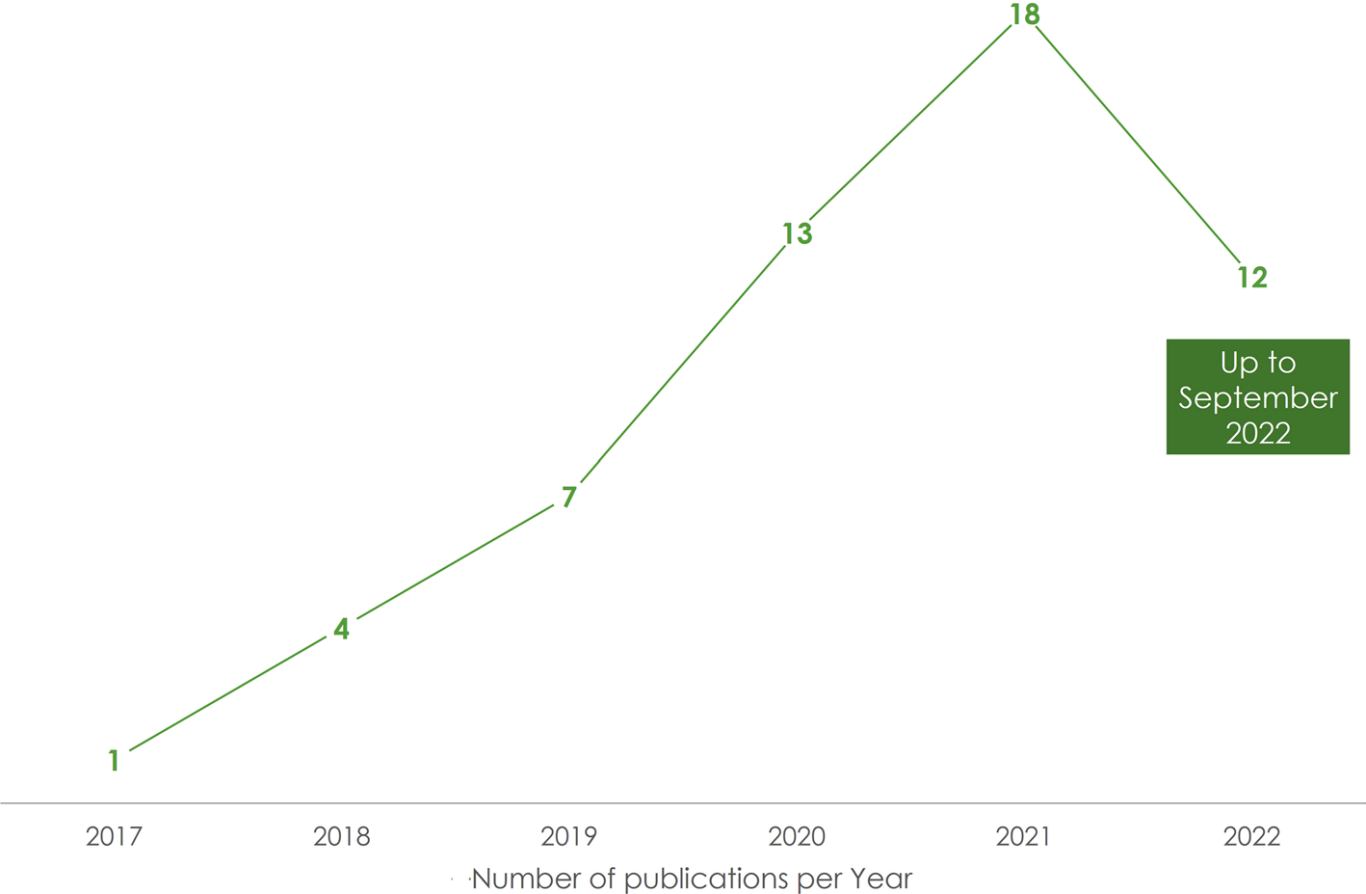
Publications per year

The research study identified 55 related publications that qualify for reporting. As illustrated in Fig. 3, there has been an increasing number of publications over the last 5 years. Starting from one publication in 2017, this number reached 17 in 2021.

**Fig. 4:**
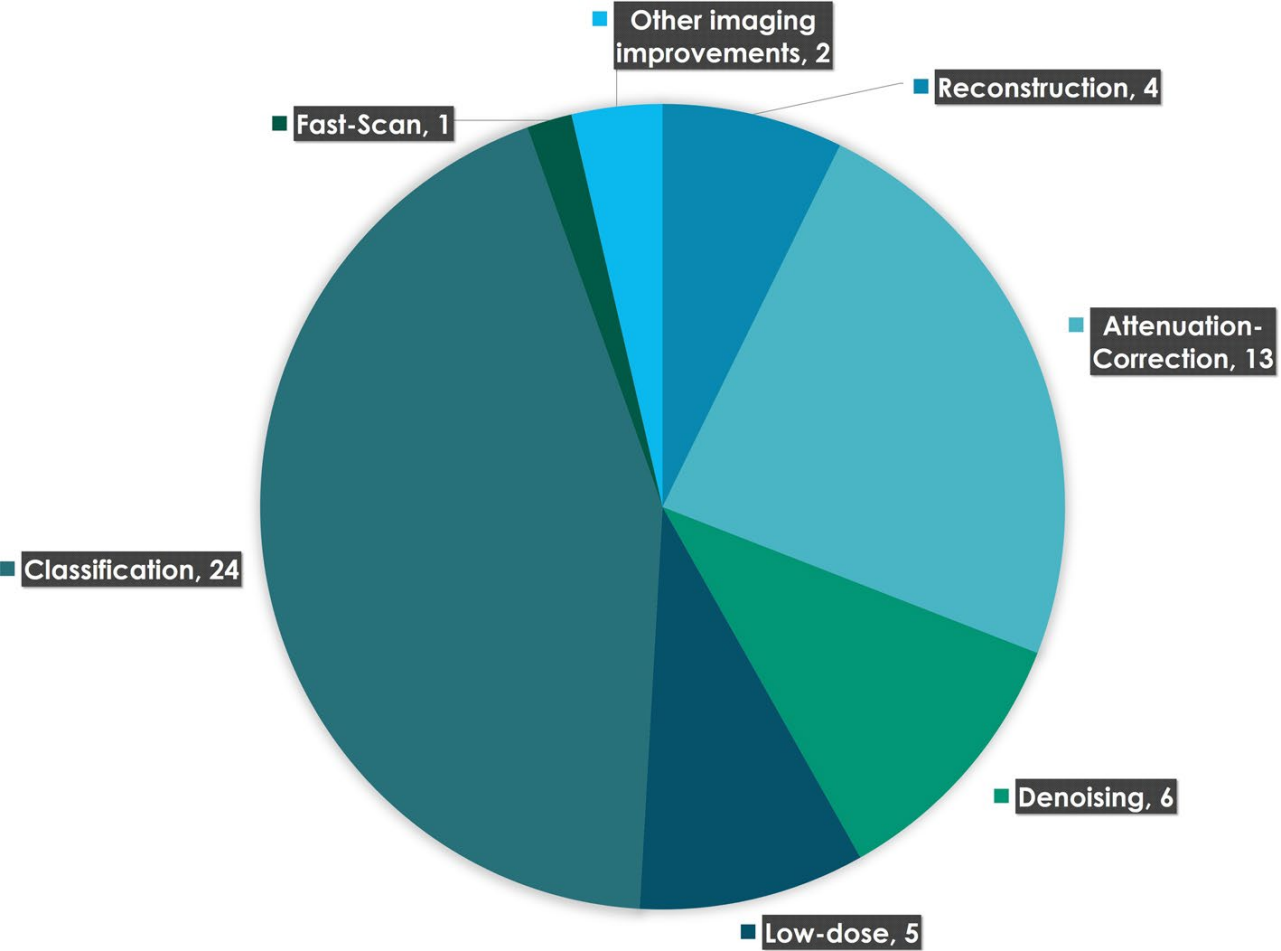
Domains of application

Five major application domains were identified during each qualified study's analysis: diagnosis/classification, denoising, full-count SPECT estimation, SPECT attenuation correction (AC), and reconstruction. The latter four domains belong to a broader spectrum: image quality improvement. The distribution of publications among these domains is presented in Fig. 4.

**Fig. 5:**
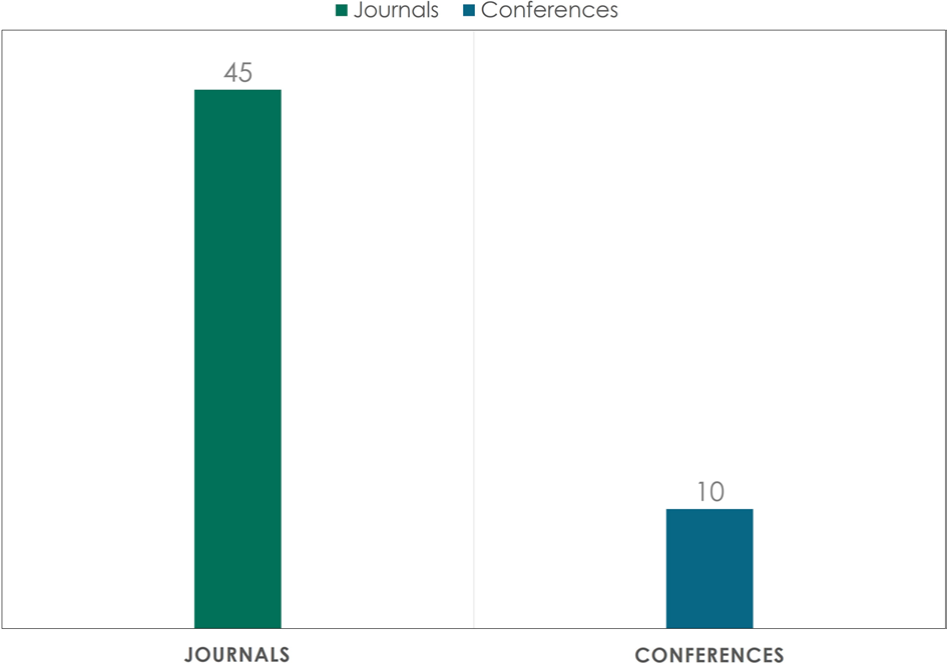
Number of publications in scientific journals and conferences

**Fig. 6:**
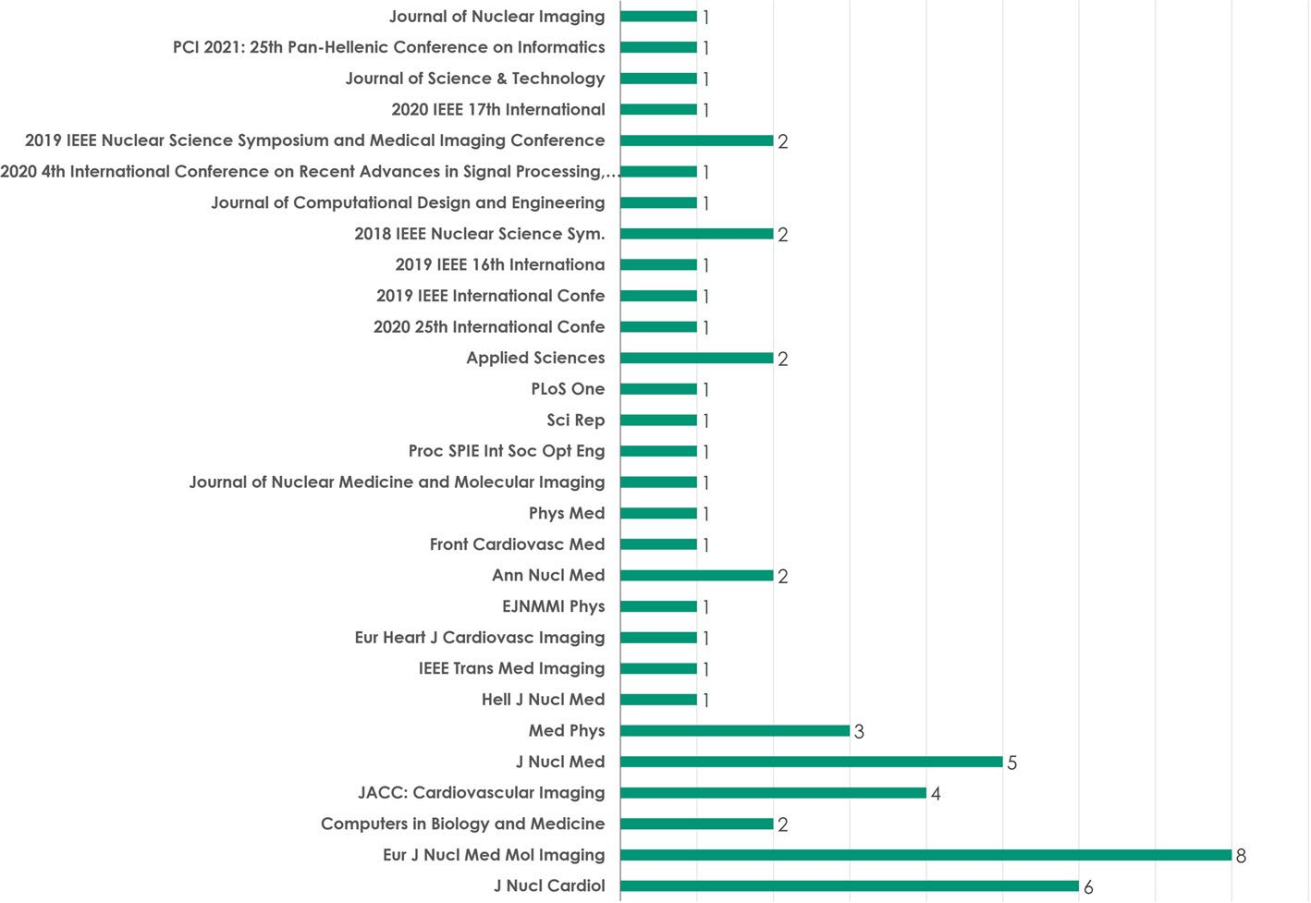
Overview of the publishing journals and conferences

The study reviewed publications presented in scientific journals and conferences. Out of the 55 reviewed publications, 45 are published in peer-reviewed journals, as illustrated in Fig. 5. An analytical overview of the journals and conferences that participate in publishing is presented in Fig. 6.

### Diagnosis/classification

Cardiovascular disease diagnosis was the most popular domain of DL application in SPECT studies. It refers to CAD diagnosis, myocardial defect identification, and abnormality detection in Polar Maps or SPECT scans. CNNs are the dominant DL strategy for this type of classification. Several research studies deploy state-of-the-art pre-trained CNNs that have already succeeded in relevant applications. In contrast, many studies develop their own CNN architectures intending to propose task-specific models that seek and extract medical image features. ML methods have also been evaluated in recent studies. ML methods differentiate themselves from the DL methods because they do not process the SPECT images directly. Instead, they analyze clinical data or predefined image features for the same task. Table 1 summarizes the presented literature regarding the diagnosis and classification tasks. Table 2 showcases studies that perform external validation of the proposed techniques.

**Table 1:** List of identified diagnosis/classification studies along with their main characteristics

No.	First author	Year	Title	Input	Learning algorithm	Reference	Outcome	Validation (total num. of images)	Explainability	Results
	Kenichi Nakajima	2017	Diagnostic accuracy of an artificial neural network compared with statistical quantitation of myocardial perfusion images: a Japanese multicenter study	SPECT	ANN	Human reader	Normal or abnormal	Hold out: 1001 training, 364 test (the test set is multicenter) (total num images: 1001)	Indirectly via segmenting the images into sub-images	AUC: 0.89–0.92 for stenosis > = 50% AUC: 0.58–0.72 for stenosis > = 75%
	Julian Betancur	2018	Deep learning for prediction of obstructive disease from fast myocardial perfusion SPECT: a multicenter study	SPECT	CNN (hand-crafted)	ICA	CAD\No-CAD and per-vessel	10FCV (1,638)	–	Per-patient sensitivity improved from 0.79 (TPD) to 0.82 (DL) Per-vessel sensitivity improved from 0.64 (TPD) to 0.69 (DL)
	Julian Betancur	2018	Prognostic value of combined clinical and myocardial perfusion imaging data using machine learning	SPECT + age, sex, and risk factors	LogitBoost	ICA	MACE risk	Stratified 10FCV (Total num images: 2619)		AUC: 0.8
	Nathalia Spier	2019	Classification of polar maps from cardiac perfusion imaging with graph-convolutional neural networks	Polar Maps	Graph CNNs (hand-crafted)	Human reader	Normal and abnormal	Hold-out for localization and 4FCV for the entire dataset (946 subjects)	Indirectly via segmenting the images into sub-images	Agreement rating (segment-by-segment): 0.83 Sensitivity: 0.47 Specificity: 0.70
	Julian Betancur	2019	Deep learning analysis of upright-supine high-efficiency SPECT myocardial perfusion imaging for prediction of obstructive coronary artery disease: a multicenter study	SPECT	CNN (hand-crafted)	ICA	CAD\No-CAD and per-vessel risk	Leave-One-Center-Out CV (4 groups, 1160 images)	–	Per-patient: AUC of 0.81 (DL) versus AUC of 0.78 (cTPD) Per-vessel: AUC of 0.77 (DL) versus AUC of 0.729 AUC (cTPD)
	Rahmani	2019	Improved diagnostic accuracy for myocardial perfusion imaging using artificial neural networks on different input variables including clinical and quantification data	Polar maps + cardiac risk factors	ANN	ICA	CAD\No-CAD	14 cases for validation (total num mages: 93)		Accuracy: 0.857
	Selcan Kaplan Berkaya	2020	Classification models for SPECT myocardial perfusion imaging	SPECT	CNN (VGG-19)	Human reader	Normal or abnormal	Training (66%), validation (17%), and test (17%) (Total num images: 192)	–	Accuracy: 0.93 Sensitivity: 1.0 Specificity: 0.86
	Yuka Otaki	2020	Diagnostic accuracy of deep learning for myocardial perfusion imaging in men and women with a high-efficiency parallel-hole-collimated cadmium-zinc-telluride camera: multicenter study	Polar Maps	CNN	ICA	CAD\No-CAD	Leave-one-center-out external validation (4 centers in total) (Total num images: 1160)	Grad-CAM	Sensitivity: DL (0.82), SSS (0.75), U-TPD (0.77) and S-TPD (0.73) in men DL (0.71), SSS (0.71), U-TPD(0.7) and S-TPD(0.65) in women
	Apostolopoulos	2020	Automatic characterization of myocardial perfusion imaging polar maps employing deep learning and data augmentation	Polar Maps	CNN (VGG-16)	ICA	CAD\No-CAD	10FCV (216 subjects)	–	DL: Accuracy of 0.74, Sensitivity of 0.75. Specificity of 0.73. Similar results with the experts Semi-Quantitative Analysis: Accuracy of 0.66
	Apostolopoulos	2020	Multi-input deep learning approach for cardiovascular disease diagnosis using myocardial perfusion imaging and clinical data	Polar Maps + Clinical	CNN (Inception V3) + Random Forest	ICA	CAD\No-CAD	10FCV (566 subjects)	–	Expert Accuracy: 0.7 Sensitivity: 0.89 Specificity: 0.71 Model Accuracy: 0.78 Sensitivity: 0.77 Specificity: 0.79 Cohen's Kappa: 72.24
	Lien-Hsin Hu	2020	Machine learning predicts per-vessel early coronary revascularization after fast myocardial perfusion SPECT: results from multicentre REFINE SPECT registry	18 clinical variables, 9 stress-test variables, and 28 imaging variables	LogitBoost	ICA	CAD\No-CAD Per-vessel and per-patient	10FCV (Total num images: 1980)		Per-vessel AUC: 0.79 Per-patient per-vessel AUC: 0.81
	Baskaran	2020	Machine learning insight into the role of imaging and clinical variables for the prediction of obstructive coronary artery disease and revascularization: an exploratory analysis of the CONSERVE study	SPECT + BMI, age, and angina severity	XGBoost	ICA	CAD\No-CAD	5FCV (total num images: 719)		AUC: 0.779
	Trung	2020	A deep learning method for diagnosing coronary artery disease using SPECT images of heart	SPECT	VGG-16	Human reader	CAD\No-CAD	5FCV (total num images: 1413)		Precision: 0.823
	Jui-Jen Chen	2021	Convolutional neural network in the evaluation of myocardial ischemia from CZT SPECT myocardial perfusion imaging: comparison to automated quantification	Gray SPECT images	3D-CNN (hand-crafted)	Not disclosed	CAD\No-CAD	5FCV (979 subjects)	Grad-CAM	Accuracy: 0.87 Sensitivity: 0.81 Specificity: 0.92
	Hui Liu	2021	Diagnostic accuracy of stress-only myocardial perfusion SPECT improved by deep learning	Stress only SPECT	Resnet-34	Human reader	Normal or Abnormal	5FCV (total num images: 37,243)	–	AUC: 0.872 ± 0.002
	Papandrianos	2021	Automatic diagnosis of coronary artery disease in SPECT myocardial perfusion imaging employing deep learning	SPECT	RGB-CNN (hand-crafted)	Human reader	Normal or Abnormal	Hold out 85–15% (244 subjects)	–	Accuracy: 0.93 ± 0.28 AUC: 0.936
	de Souza Filho	2021	Machine learning algorithms to distinguish myocardial perfusion SPECT polar maps	SPECT	Random forest	Human reader	Normal or Abnormal	10FCV (total num mages: 1007)		AUC: 0.853 Accuracy: 0.938 Precision: 0.968 Sensitivity: 0.963
	Arvidsson	2021	Prediction of obstructive coronary artery disease from myocardial perfusion scintigraphy using deep neural networks	Polar maps + angina symptoms and age	CNN	ICA	Probability of CAD in the left anterior artery left circumflex artery and right coronary artery	5FCV (total num images: 760)		Per-vessel AUC: 0.89 Per-patient AUC: 0.95
	Zahiri	2021	Deep learning analysis of polar maps from SPECT myocardial perfusion imaging for prediction of coronary artery disease	Polar maps	CNN	Human reader	Normal or abnormal	5FCV (total num images: 3318)		Accuracy:0.7562 Sensitivity:0.7856 Specificity: 0.7434 F1 score: 0.6646 AUC: 0.8450
	Papandrianos	2022	Exploring classification of SPECT MPI images applying convolutional neural networks	SPECT	CNN (hand-crafted)	Human reader	Normal or abnormal	87 cases for validation (Total num images: 314)	–	Accuracy: 0.90 AUC: 0.937
	Papandrianos	2022	Deep learning exploration for SPECT MPI polar map images classification in coronary artery disease	Polar maps	RGB-CNN	Human reader	Normal and abnormal	5FCV (Total num images: 314)		Accuracy: 0.92
	Miller	2022	Explainable deep learning improves physician interpretation of myocardial perfusion imaging	SPECT	DL model	ICA	CAD\No-CAD	(Total num images: 240)	Explainability	When the readers use DL AUC: 0.779 When the readers do not use DL AUC: 0.747 When DL runs autonomously AUC: 0.793
	Ananya Singh	2022	Direct risk assessment from myocardial perfusion imaging using explainable deep learning	Stress rest polar maps combined with age, sex, and cardiac volumes	CNN (hand-crafted)	ICA	Prediction of death or nonfatal myocardial infarction (MI)	10FCV (20,401 subjects)	Grad-CAM	AUC: 0.76 (internal) AUC: 0.73 (external)

**Table 2:** Classification studies performing external validation

No.	First author	Title	Type of external testing	Number of patients	Results
1	Singh	Direct Risk Assessment From Myocardial Perfusion Imaging Using Explainable Deep Learning	External testing group	20,401 patients in the training and internal testing group (5 sites) and 9,019 in the external testing group (2 different sites)	AUC: 0.73
2	Betancur	Deep Learning for Prediction of Obstructive Disease From Fast Myocardial Perfusion SPECT A Multicenter Study	Multicenter	Total of 1,638 patients (67% men) without known coronary artery disease, undergoing stress 99mTc-sestamibi or tetrofosmin MPI with new generation solid-state scanners in 9 different sites	Per patient AUC: 0.80 Per vessel: AUC: 0.76
3	Nakajima	Diagnostic accuracy of an artificial neural network compared with statistical quantitation of myocardial perfusion images: a Japanese multicenter study	Multicenter	364 patients collected from nine hospitals served as the validation dataset	Stress defects AUC: 0.92 Stress-induced ischemia AUC: 0.90 for patients with old myocardial infarction based on rest defects AUC: 0.97
4	Otaki	Clinical Deployment of Explainable Artificial Intelligence of SPECT for Diagnosis of Coronary Artery Disease	Multicenter	External testing was performed in 555 patients from 2 centers	AUC: 0.83
5	Apostolopoulos	Multi-input deep learning approach for Cardiovascular Disease diagnosis using Myocardial Perfusion Imaging and clinical data	Image acquisition device variation	98 patients	Accuracy: 79.16%
6	Hu	Machine learning predicts per-vessel early coronary revascularization after fast myocardial perfusion SPECT: results from multicentre REFINE SPECTregistry	Multicenter	1980 patients from 9 centres	Per-vessel AUC: 0.79 Per-patient AUC: 0.81
7	Betancur	Deep Learning Analysis of Upright-Supine High-Efficiency SPECT Myocardial Perfusion Imaging for Prediction of Obstructive Coronary Artery Disease: A Multicenter Study	Division of dataset in 4 groups and performed a leave one-center-out cross-validation for each center. Overall predictions for each center were merged to have an overall estimation of the multicenter performance	1160 patients	Per-patient AUC: 0.81 Per-vessel AUC: 0.77
8	Otaki	Diagnostic Accuracy of Deep Learning for Myocardial Perfusion Imaging in Men and Women with a High-Efficiency Parallel-Hole-Collimated Cadmium-Zinc-Telluride Camera: multicenter study	Training and testing datasets included both men and women for prediction of obstructive CAD using repeated leave-one-center-out external validation (4 models built from 3 centers and tested in 4th center)	1160 patients in 4 separate centers	Sensitivity: 82% in men, and 71% in women

#### Handcrafted CNNs

Various studies propose handcrafted CNNs that distinguish between normal and abnormal SPECT images visualizing the myocardia, or Polar Maps, which summarize the 3D information of multiple heart views into a single polar plot.

Papandrianos et al. [18] presented an RGB-CNN model to classify SPECT images concerning their abnormal findings. There are a total of 513 cases, and they are represented in stress and rest conditions. The problem that is addressed is the differentiation of normal and ischemic images. Data scarcity issues were circumvented by applying data augmentations. The proposed model accomplished 90.2% accuracy and a 93.77% AUC value in discriminating ischemic from normal SPECT images, with the human reader interpretation considered as the ground truth. These results demonstrate the magnificent capability of the model to predict correctly, despite the small dataset. The same research team [19] extended their previous work to diagnose ischemia and/or infarction using CNNs. The dataset of the corresponding research includes a total of 224 patients who had undergone stress and rest SPECT tests. The participants underwent invasive coronary angiography (ICA) 40 days after MPI. Two DL techniques were followed: an (a) implementation of RGB-CNN from scratch and (b) transfer learning to classify images as normal or abnormal. The pre-trained models were VGG16, DenseNet, MobileNet, and InceptionV3. With reference to the visual assessment performed by medical experts, the results reported significant abilities of the proposed CNN with an overall accuracy of 93.48 ± 2.81%. This accuracy is significantly improved compared to the 90.2%, which was initially obtained in [18].

Narges Zahiri et al. [20] aimed to explore the potential of deep CNNs to distinguish between normal and abnormal polar maps with reference to the physician’s diagnosis. The dataset included 3318 stress and rest polar maps. Data augmentation was utilized to expand the training dataset. The proposed DL model was thoroughly validated under a fivefold cross-validation procedure. The model achieved a 0.845 AUC. Besides, the inclusion of rest perfusion maps significantly improved the AUC of the DL model (AUC: 0.845) compared with stress polar maps only (AUC: 0.827). Papandrianos et al. [21] explored the potential of automatic classification of polar maps between normal and abnormal by implementing a custom RGB CNN. The study included 314 polar maps in stress, rest representation, and AC and NAC formats. RGB-CNN was trained using physician interpretation as ground truth. The RGB-CNN proposal competed against the pretrained VGG-16 network. According to the results, RGB extracted 92.07% and VGG-16 95.83%. RGB-CNN competed against robust state-of-the-art methods.

Some research compares DL-based results against quantifiable metrics advised by the guidelines. For example, Yuka Otaki et al. [22] developed a DL model to identify CAD and compared its results against the Total Perfusion Deficit (TPD) method. One thousand one hundred sixty patients were included to classify raw upright and supine stress MPI polar maps. MPI and ICA were performed within a 6-month interval. As an external validation method, leave-one-centre-out was utilized with four models. Julian Betancur et al. [23] designed a CNN for the same purpose. The number of participants was 1160, whilst the utilized data involved semi-upright and supine stress Polar Map representations. The classification of obstructive disease was evaluated using the leave-one-centre-out cross-validation technique with four centres, where all validated predictions were merged to avoid a calculation for a single centre. The CNN model performs the diagnosis without adding predefined coronary territories. The performance of CNN was compared against combined perfusion quantification by TPD, achieving 84.8% sensitivity versus 82.6% obtained with clinical reading. In a subsequent study, Julian Betancur et al. [24] evaluated the automatic diagnosis of CAD from SPECT image inputs in contrast with TPD with a deep CNN. A total of 1638 patients without known CAD and with ICA performed within 6 months of MPI were examined. The data involved raw and quantitative polar maps in only stress representation. A stratified tenfold cross-validation procedure was adopted. The AUC score for disease prediction by their proposed DL scheme was superior to TPD (per patient: 0.80 vs 0.78; per vessel: 0.76 vs 0.73). With the DL threshold set to the same specificity as TPD, per-patient sensitivity improved from 79.8% (TPD) to 82.3%, and per-vessel sensitivity improved from 64.4% (TPD) to 69.8%.

Besides distinguishing between normal and abnormal subjects, some studies aim to perform region-based classification. Arvidsson et al. [25] developed a CNN to predict obstructive coronary artery disease in the left anterior artery, left circumflex artery, and right coronary artery using SPECT Polar Maps. A total of 588 patients were included in this study, whilst clinical data like angina symptoms and age were also utilized. The proposed CNN framework achieved an average AUC of 0.89 per vessel and 0.95 per patient, using the ICA findings as a reference. Furthermore, gradient-weighted class activation mapping (Grad-CAM) was utilized to visually demonstrate the regions on which predictions are based to extract the output. The authors observed sex differences in the diagnostic performance of DL for the prediction of obstructive CAD from D-SPECT, with DL outperforming visual and TPD in men but not in women.

An increasing number of works propose explainable DL-based methods that perform image classification and inform the user about the suggested areas of interest wherein the model bases its predictions. Miller et al. [26] utilized an explainable DL model to improve the diagnostic accuracy of CAD and aid physical interpretation. A total of 240 patients underwent MPI examinations and were included in this study, with ICA as a reference. Regarding the results, human readers using the DL’s prediction achieved an AUC of 0.779, whereas their interpretation without DL reached an AUC of 0.747. It is worth mentioning that DL, on its own, achieved an AUC of 0.793. Yuka Otaki et al. [27, 28] proposed an explainable DL model to detect obstructive CAD. A total of 3578 patients with suspected CAD from 9 centres were enrolled. The authors proposed a hand-crafted CNN to process the SPECT Polar Maps in stress conditions. In the fully-connected layer of the CNN, the authors supplied the sex and age of the patient to increase the number of features. Concerning ICA findings, this method achieved an Area Under Curve (AUC) score of 0.83 following a tenfold cross-validation procedure, which was superior to the quantitative analysis results by expert readers (AUC = 0.8). Also, attention maps were produced to highlight the regions and segments contributing most to the per-vessel prediction.

Singh et al. [29] developed an explainable deep learning model to predict nonfatal myocardial infarction (MI) or death, which also provides highlighted image regions related to obstructive CAD. The study included 20,401 patients, who went under SPECT MPI procedure for training and internal testing purposes and 9019 patients were added from external testing group gathered from two different sites. The external testing group was included to evaluate generalizability. The dataset consisted of polar maps in stress and rest representation with the inclusion of age, sex and cardiac volumes, which were added at the first fully connected layer. Referring to explainability, Grad-CAM was developed. For comparison reasons a logistic regression model was developed with the following values age, sex, stress total perfusion deficit (TPD), rest TPD, stress left ventricular ejection fraction, and stress left ventricular end-systolic volume. The model achieved and AUC of 0.76, which is higher than stress TPD with 0.63 AUC, ischemic TPD with 0.6 AUC and compared to logistic regression model, which extracted 0.72 AUC. The developed model improved accuracy in contrast to traditional quantitative approaches and is well calibrated and provides robust results.

Jui-Jen Chen et al. [30] examined 979 SPECT subjects from a local hospital for the diagnosis. However, whether the images are labelled based on experts' visual inspection or on the ICA's findings is not reported. A three-dimensional CNN has been applied to classify the SPECT slices. Furthermore, Grad-CAM heat maps have been produced to identify myocardial defects in the images. The proposed model obtained accuracy, sensitivity, and specificity metrics of 87.64%, 81.58%, and 92.16%, respectively, in distinguishing between normal and abnormal images using a test set of 89 samples. Nathalia Spier et al. [31] investigated Graph CNNs for CAD diagnosis. They enrolled 946 polar map images in stress and rest representations of the heart. Labelling has been done using the human observer interpretation. Also, heatmaps were produced and demonstrated the segments of the heart that were indicated as pathological. The extracted results demonstrate adequate performance in classifying unseen data under a fourfold cross-validation procedure, in contrast to clinical visual analysis, with 92.8% and 95.9% specificity in rest and stress data, respectively. The proposed model achieves an agreement with the human observer on 89.3% of rest test polar maps and on 91.1% of stress test polar maps. Localization performed on a fine 17-segment division of the polar map achieves an agreement of 83.1% with the human observer.

#### CNNs and transfer learning

Selcan Kaplan Berkaya et al. [32] intended to produce a classification model to classify SPECT images and identify perfusion abnormalities like ischemia and infarction. The summed stress and rest images from 192 patients were studied. Two models were proposed. The first is a DL-based model which employs State Of The Art (SOTA) CNNs and fully-connected layers of support vector machines (SVM) for the classification of the deep extracted image features. As far as the second model is concerned, it involves image processing techniques like segmentation, feature extraction, and colour thresholding applied to segmented parts of each SPECT slice. This method extracts five predefined image features classified by a rule-based algorithm. With reference to the visual assessment as performed by the experts, the integrated CNN-SVM model achieved 92% accuracy, 84% sensitivity, and 100% specificity, whereas the knowledge-based classification attained 93% accuracy, 100% sensitivity, and 86% specificity. Those metrics are reported on a test dataset that includes 17% of the total samples. Hui Liu et al. [33] demonstrated a DL approach to automatically diagnose myocardial perfusion abnormalities in abnormal and normal with only stress MPI profile maps as input. A total of 37,243 patients who underwent stress-only and stress/rest SPECT MPI have been examined. The study involved three SPECT/CT cameras. There was an addition of six extra features, including gender, BMI, length, stress type, radiotracer, and the option of including or not including the attenuation correction. The ResNet-34 model is employed to perform the feature extraction. The results were compared against the conventional quantitative perfusion defect size (DS) method. With reference to the diagnostic impression from nuclear cardiologists, the model achieved an AUC of 0.87, outperforming the DS method. Also, the proposed network showed robustness to image acquisition device variation, achieving an 82% and 84% accuracy in all scanners. The model also achieved greater performance in female participants, reaching an accuracy of 87%.

Apostolopoulos et al. [34] used the Polar Map images under stress and at rest to diagnose CAD using the pretrained VGG16 model. The study involved 216 participants. The attenuation correction (AC) and the non-attenuation correction (NAC) Polar Map images were merged into a single image per patient. With reference to the findings of ICA, VGG16 achieved an accuracy of 74.53%, a sensitivity of 75.00% and a specificity of 73.43%. The respective figures for MPI interpretation by experienced nuclear medicine physicians were 75.00%, 76.97%, and 70.31%. The accuracy of semi-quantitative polar map analysis was lower, at 66.20% and 64.81% for the AC and NAC techniques, respectively. Besides, the model showed robustness to acquisition device variation. The same author team extended their study [35] by proposing a hybrid CNN-Random Forest approach for classifying Polar Map images and clinical attributes into normal and abnormal classes, using ICA findings as a reference for CAD disease. The study involved 566 patient cases. The authors used the InceptionV3 pretrained model to predict the class of the input Polar Maps. The model's output was considered a unique attribute among 22 clinical factors, such as gender and age. The Random Forest classifier was employed to predict the outcome. With reference to ICA results, the model achieved 78.44% accuracy, 77.36 sensitivity, and 79.25% specificity. The human cognitive process's overall accuracy reached 79.15%, which is approximately 1% better than the automatic model's accuracy (78.43%). Besides, the overall agreement rating between the human experts and the model was 86% (Cohen’s Kappa = 72.24). The model was also tested on unseen data from a different SPECT scanner and achieved consistent results (76.53% accuracy).

Trung et al. [36] proposed a CNN to diagnose CAD. The authors utilized polar maps and SPECT slices. The dataset included 1413 heart SPECT images labelled by a nuclear expert as CAD and non-CAD. The DL network’s (VGG-16) performance was evaluated using fivefold cross-validation. The results indicated that SPECT images guarantee a better diagnosis than polar maps, with a precision of 86.14% ± 2.14% and 82.57% ± 2.33%, respectively.

#### Machine learning

Kenichi Nakajima et al. [37] proposed an ANN for CAD diagnosis and myocardial ischemia and/or infarction detection. This research consisted of 1001 stress/rest MPI images for training, and there was an addition of 364 images for validation. Expert interpretations served as the gold standard. The achieved results were compared against the conventional quantitative approach. The ANN algorithm outperformed the conventional summed difference scores, scoring an AUC of 0.92 in identifying stress defects and 0.91 in stress-induced ischemia.

Souza Filho et al. [38] explored the potential of developing different ML models like Adaptive Boosting (AB), Gradient Boosting (BG), Random Forest (RF), and Extreme Gradient Boosting (XGB) to find the ideal model for efficient differentiation between normal and abnormal cases of SPECT Polar Maps labelled by human readers. The stress and rest conditions included a total of 1007 Polar Maps. Each image was divided into five horizontal and five vertical slices, where the sum of pixel intensities from each slice was computed, and ten attributes were acquired. Afterwards, data augmentation was applied to generate 324 Polar Maps. RF was concluded to have the best sensitivity with 96%, whereas AB, GB, and XGB obtained 92%, 94%, and 95%, respectively.

Hu et al. [37] evaluated the per-vessel and per-patient predictions using an ML methodology. A total of 1980 patients were utilized in stress and rest demonstrations, and overall, 18 clinical, nine stress test and 28 imaging variables were utilized for this study. The model achieved an AUC of 0.79 on a patient-level basis and 0.81 on a vessel-level basis using ICA findings for reference. Baskaran et al. [39] investigated the importance of including clinical and imaging variables for the successful prediction and revascularization of CAD by developing XGBoost and estimated the results by developing a fivefold cross-validation. Seven hundred nineteen ICA-confirmed patients were included in this research. The proposed model performed similarly to previous history-based scores and achieved an AUC of 0.779, a sensitivity of 89.2%, and a specificity of 92.9%. Following the results, BMI is the most valued non-imaging variable to be included in the prediction and revascularization of CAD. Nevertheless, BMI, age, and angina severity are the most important parameters for prediction.

Betancur et al. [40] evaluated the inclusion of clinical and SPECT MPI data to predict MACE (Major Adverse Cardiac Events) by developing an ensemble boosting algorithm, LogitBoost. A total of 2617 patients were considered under stress examination. Twenty-eight clinical variables, seventeen stress test variables, and twenty-five imaging variables were included. Furthermore, LogitBoost with both clinical and imaging data (ML-Combined) was compared against the utilization of only imaging variables as input, and visual diagnosis and automated quantitative imaging analysis, and ML-combined outperformed with an AUC of 0.81. Rahmani et al. [41] aimed to investigate the integration of ANN to predict obstructive CAD by adding clinical data. Ninety-three polar maps were included, with the patients in stress and rest demonstrations. Regarding the clinical data, various combinations were examined, and the accuracy increased with age, gender, and the number of cardiac risk factor additions. ANN achieved 85.7% accuracy and improved the results by adding patient data.

### Image quality improvement

Image quality improvement alludes to various improvements, including low-count SPECT estimation, AC, de-noising, and reconstruction. Table 3 presents the reviewed literature.

**Table 3:** List of identified image quality improvement studies along with their main characteristics

No.	First author	Year	Title	Input	Learning algorithm	Outcome	Validation (total num. of images)	Results
*Low-dose*								
1	Ramon	2018	Initial investigation of low-dose SPECT-MPI via deep learning	SPECT: Low-dose images (1/8 dose)	3D-CNN	SPECT: Full-dose images	(Total num images: 930)	FBP: 0.99 O-SEM: 0.99
2	Olia	2021	Deep learning–based denoising of low‑dose SPECT myocardial perfusion images: quantitative assessment and clinical performance	SPECT: Low-dose images (½ dose)	Generative adversarial network	SPECT: Full-dose images	35 patients as an external dataset (Total num images: 345)	PSNR: 42.49 ± 2.37 SSIM: 0.99 ± 0.01 RMSE: 1.99 ± 0.63 R: 0.997 ± 0.001
3	Ramon	2020	Improving diagnostic accuracy in low-dose SPECT myocardial perfusion imaging with convolutional denoising networks	SPECT: Low-dose images (½ dose)	3D CNN	SPECT: Full-dose images	122 patients for validation (Total num images: 1052)	AUC: 0.799
4	Song	2019	LOW-DOSE CARDIAC-GATED SPECT STUDIES USING A RESIDUAL CONVOLUTIONAL NEURAL NETWORK	SPECT: Low-dose images (¼ dose)	3D residual convolutional neural network (CNN	SPECT: Full-dose images	12 patients for validation (Total num images: 119)	NMSE = 0.153
5	Song	2020	Low-dose cardiac-gated spect via a spatiotemporal convolutional neural network	SPECT: Low-dose images (¼ dose)	Spatiotemporal CNN	SPECT: Full-dose images	12 cases for validation (Total num images: 119)	(NMSE = 0.273)
*Fast-Scan*								
6	Shiri	2020	Standard SPECT myocardial perfusion estimation from half-time acquisitions using deep convolutional residual neural networks	SPECT: Fast scan images	ResNet	SPECT: Standard time scan	10FCV (Total num images: 363)	RMSE: 6.8 ± 2.7 ARE: = 3.1 ± 1.1% SSIM: = 0.97 ± 1.1 PSNR: = 36.0 ± 1.4
*Attenuation-correction*								
7	Yang	2021	Direct attenuation correction using deep learning for cardiac SPECT: a feasibility study	SPECT: NAC	DCNN	SPECT: AC	10FCV (Total num images: 100)	Mean ± SD: 0.49% 6 4.35% Average absolute segmental errors: 3.31% 6 2.87%
8	Shi	2020	Deep learning-based attenuation map generation for myocardial perfusion SPECT	Both photopeak window and scatter window SPECT images + gender, age, height, weight and body mass index (BMI)	Deep fully convolutional neural networks	Attenuation maps	25 testing images (Total num images: 65)	The NMAE between the reconstructed SPECT images that were corrected using the synthetic and CT-based attenuation maps was 0.26% ± 0.15%, whereas the localized absolute percentage error was 1.33% ± 3.80% in the left ventricle (LV) myocardium and 1.07% ± 2.58% in the LV blood pool
9	Mostafapour	2021	Deep learning-based attenuation correction in the image domain for myocardial perfusion SPECT imaging	SPECT: NAC	ResNet-U-Net	Attenuation maps	19 SPECT non-AC images as external validation and 5% oc training was dedicated as validation dataset (Total num images: 99)	ME ResNet: 6.99 ± 16.72 SSIM ResNet: 0.99 ± 0.04 ME U-Net: − 4.41 ± 11.8 SSIM U-Net: 0.98 ± 0.05
10	Chen	2021	Direct and indirect strategies of deep-learning-based attenuation correction for general purpose and dedicated cardiac SPECT	Both photopeak and scatter windows	U-Net and DuRDN	μ-maps	20 cases for validation (Total num images: 270)	NMSE: 1.20 ± 0.72%
11	Liu	2021	Post-reconstruction attenuation correction for SPECT myocardial perfusion imaging facilitated by deep learning-based attenuation map generation	SPECT: NAC	3D GAN	Attenuation maps	30 cases for validation (Total num images: 30)	Global nMAE: 2 1.3% ± 8.0% Localized absolute percentage errors: 2 3.8% ± 4.5%
12	Hagio	2022	“Virtual” attenuation correction: improving stress myocardial perfusion SPECT imaging using deep learning	SPECT: NAC maps	based on U-Net	DLAC attenuation-corrected polar maps	1865 for validation (Total num images: 11,532)	Test1: Sensitivity improved by 18.9% for DLAC accuracy improved by 13.1% Test2: specificity improved by 8% and accuracy by 5.5%
13	Chen	2021	CT-free attenuation correction for dedicated cardiac SPECT using a 3D dual squeeze-and-excitation residual dense network	Hybrid SPECT/CT stress/rest + BMI, gender, and scatter-window	3D Dual Squeeze-and-Excitation Residual Dense Network (DuRDN)	SPECT AC	40 for validation (Total num images: 172)	nRMSE: 2.01 ± 1.01%,
14	Torkaman	2021	Direct image-based attenuation correction using conditional generative adversarial network for SPECT myocardial perfusion imaging	SPECT: NAC	3D cGAN	Attenuation maps	5FCV (Total num images: 100)	nRMSE: 0.1410 ± 0.0768
15	Chen	2022	Cross-vender, cross-tracer, and cross-protocol deep transfer learning for attenuation map generation of cardiac SPECT	SPECT: NAC	DuRDN	SPECT AC	30 studies for validation (Total num images: 120)	nME: 1.11 ± 1.57%
16	Mostafapour	2022	Deep learning-guided attenuation correction in the image domain for myocardial perfusion SPECT imaging	SPECT: NAC	ResNet-U-Net	SPECT AC	19 patients for external validation (Total num images: 99)	ResNet and U-Net models resulted in an ME of − 6.99 ± 16.72 and − 4.41 ± 11.8 and an SSI of 0.99 ± 0.04 and 0.98 ± 0.05
17	Nguyen		3D U-Net generative adversarial network for attenuation correction of SPECT images	SPECT: NAC	3DU-Net-Gan	SPECT AC	279 studies for validation (Total num images: 603)	SSIM: 0.945% nME: 0.034
18	Shanbhag	2022	Deep learning-based attenuation correction improves diagnostic accuracy of cardiac SPECT	Short-axis NC and AC images	cGAN	SPECT: Full-dose images	Total num images: 5490	AUC: 0.79 Normalcy rate: 70.4%
19	Hagio	2021	“Virtual” attenuation correction: improving stress myocardial perfusion SPECT imaging using deep learning	NAC	U-Net	Attenuation–corrected (DLAC) perfusion polar maps	Total num images: 11,532	AUC: 0.827
*Denoising*								
20	Liu	2020	Deep learning with noise-to-noise training for denoising in SPECT myocardial perfusion imaging	SPECT	CU-Net	Denoised images	121 for validation (Total num images: 895)	PSNR was improved on average by 23%
21	Liu	2021	Improving detection accuracy of perfusion defect in standard dose SPECT-myocardial perfusion imaging by deep-learning denoising	SPECT	3D CU-Net	Denoised images	121 for validation (Total num images: 190)	AUC: 0.86
22	Kikuchi	2022	A myocardial extraction method using deep learning for 99mTc myocardial perfusion SPECT images: A basic study to reduce the effects of extra-myocardial activity	SPECT	U-Net++	Myocardial region extraction	71 cases for validation (Total num images: 694)	Dice coefficient: 0.918
23	Sun	2022	Dual gating myocardial perfusion SPECT denoising using a conditional generative adversarial network	SPECT	3D cGAN	Denoised images	6 cases for validation (Total num images: 20)	Best resolution with less blurring: 0.1671 ± 0.078
24	Sun	2019	Generative adversarial network for denoising in dual gated myocardial perfusion SPECT using a population of phantoms and clinical data	SPECT/CT	GAN	Denoised images	(Total num images: 5 4D XCAT phantoms)	NSD:0.083
25	Mok	2018	Initial investigation of using a generative adversarial network for denoising in dual gating myocardial perfusion SPECT	SPECT	GAN	Denoised images	(Total num images: 1152 frames)	NSD: 0.093
*Reconstruction*								
26	Song	2019	Approximate 4D reconstruction of cardiac-gated spect images using a residual convolutional neural network	SPECT	3D residual CNN	SPECT reconstructed images	20 cases for validation (Total num images: 197)	NMSE = 0.033
27	Xie	2022	Increasing angular sampling through deep learning for stationary cardiac SPECT image reconstruction	4D extended cardiac-torso phantoms heart images	U-Net	Generated synthetic four-angle images	(Total num images: 8 pig studies and two physical phantoms, and 20 human studies)	SD: 0.68
28	Dietze	2019	Accelerated SPECT image reconstruction with FBP and an image enhancement convolutional neural network	SPECT/CT scans	CNN	SPECT reconstructed images	20 cases for validation (Total num images: 128)	CNR: 12.5
29	Chrysostomou	2020	SPECT imaging reconstruction method based on deep convolutional neural network	Sinograms 192 × 128 × 1	CNN	Original ”true” activity the size of 128 × 128 × 1	60,000 for validation (Total num images: 600,000 phantoms)	Medium Noise: MSE: 0.003 MAE: 0.023 SSIM: 0.938 PCC: 0.971
*Other imaging improvements*								
30	Cheng	2022	Super-resolution reconstruction for parallel-beam SPECT based on deep learning and transfer learning: a preliminary simulation study	LR sinogram phantoms images	Residual network and transfer learning	High Resolution reconstructed SPECT images	three sets of XCAT phantoms (Total num images: 100,000 Shepp Logan-like phantoms, 100,000 Derenzo-like phantoms, and 100,000 Jaszczak-like phantoms)	SSIM: 0.9777
31	Zhang	2021	A novel deep-learning-based approach for automatic reorientation of 3D cardiac SPECT images	SPECT/CT	STN (spatial transformer network)	Cardiac SPECT reorientation	676 cases for validation (Total num images: 6762)	Center A R^2: 0.9926 Center B: R^2: 0.9823

#### Low-count SPECT image estimation

Reduction of human body radiation exposure is highly desirable. Reducing radiation exposure involves a low-dose SPECT scan with low-count emission data. Full-count SPECT image outcome is difficult to estimate from low-count data based on the existing image processing and de-noising methods. Besides, the low-count SPECT noise is different from the full-count noise. Fast-scan is another way to reduce radiation exposure and the patients’ discomfort during the examination. Patient pain and discomfort are responsible for artefacts due to motion. Fast scan results in low-count SPECT images. DL methods address such issues and estimate the full-count SPECT scans given the low-dose or fast-scan outcome.

A. Low-dose

Ramon et al. [42] proposed a 3D CNN based on CAEs to estimate the standard-dose SPECT image from the low-dose image. The study included 930 SPECT scans simulated at 1/8 and 1/16 of the standard clinical dose. The authors evaluated their method using the average correlation between the estimated and standard dose images. Also, the estimated images were compared to those obtained from conventional image de-noising methods (spatial post-filtering). When estimating the standard dose from 1/16 dose, the proposed method achieved similar image quality to the quality obtained from $${\raise0.7ex\hbox{$1$} \!\mathord{\left/ {\vphantom {1 8}}\right.\kern-0pt} \!\lower0.7ex\hbox{$8$}}$$ dose with conventional de-noising. In another study by Olia et al. [43], the authors explored the results of predicting the standard-dose image from a low-dose setup at half, quarter, and one-eighth dose levels. The study involved 345 patients. A GAN architecture was deployed to decrease the administered activity, ensuring stable accuracy and clinical values to achieve the standard dose image estimation. With reference to the actual standard dose images, the highest PSNR and SSIM and lowest RMSE were attained at a half-dose level. Overall, the proposed network can increase the quality of high low-dose SPECT images with 100% acceptance, according to a nuclear medicine specialist.

Ramon et al. [44] investigated the application of different 3D DL methodologies to suppress noise in low-dose SPECT MPI images. The dataset includes 1052 patients, and two reconstruction methods were applied, namely FBP (Filtered Back-Propagation) and OSEM (Ordered-Subsets Expectation–Maximization). The authors trained the model with low-dose acquisitions as input and full-dose images as target and explored different numbers of dose levels (1/2, ¼, 1/8 and 1/16 of full dose). Reviewing the results, the proposed DL approach can reduce substantial noise and enhance the accuracy compared to conventional reconstruction filtering. More specifically, with ½ dose, the model achieved 0.799 AUC, whereas full dose attained 0.801 AUC.

Similarly, Song et al. [45] explored a de-noising methodology based on a three-dimensional residual CNN for low-dose cardiac-gated SPECT images. The study includes 119 clinical cases in total. Regarding the model's training, the CNN utilized as a training dataset included the low-dose images with a 25% reduction of radiation dose as input and the corresponding full-dose images as output. The proposed CNN methodology was compared against traditional methods based on the ST-NLM (SpatioTemporal Non-Local Means) technique, and CNN attained an nMSE of 0.153, where ST-NLM and Gaussian post-filter extracted 0.163 and 0.172. Furthermore, the CNN decreased the nMSE by 6.13% and the ST-NLM, Gaussian post-filter, reduced it by 6.13%. Overall, the proposed CNN enhanced the noise reduction in the reconstructed myocardium and the spatial resolution of the LV wall.

Song et al. [46] developed a spatiotemporal CNN (ST-CNN) model for image denoising in low-dose cardiac gated SPECT studies. A total of 119 cases were included, and the proposed model is trained with low-dose images as input and includes full-dose images as output. Moreover, the authors included in the developed model an LSTM component in order to perform correctly with the format of a gated sequence. The corresponding model was compared against spatial-only S-CNN and ML reconstruction, where ST-CNN outperformed with NMSE 0.127, and S-CNN and ML extracted 0.161 and 0.273, respectively.

B. Fast-scan

Estimating the standard acquisition time image given a fast scan is seldom investigated in the literature. In the only work discovered, Shiri et al. [17] aimed to reduce the acquisition time of acquiring SPECT images from patients by exploring two approaches. The first approach refers to the reduction of scanning time per projection. The second approach refers to reducing the number of acquired projection images during acquisition. The study includes 363 cases with normal patients but various heart disorders, like infarction and ischemia, where the SPECT data were reconstructed with the OSEM algorithm. For each patient, four datasets were produced: FT (full-time projections), HT (half-time acquisition per projection), FP (full projections) and HP (half projections). The proposed method is applying a residual network, namely ResNet, to predict FT from HT and FP from HP images, and the results were evaluated with tenfold cross-validation. According to the results, the predicted FT had better image quality than the predicted FP, with a decreasing RMSE of 8.0 ± 3.6 and 6.8 ± 2.7 for FT and FP, respectively.

Moreover, the HP reconstructed images acquired better quality than the HT reconstructed images. The error increases as acquisition time is reduced. The deep neural network can effectively restore image quality.

#### Attenuation correction

The majority of dedicated cardiac SPECT scanners do not have integrated CT technology. As a result, attenuation correction (AC) for image quantification is very challenging due to the presence of artefacts. Several research papers address this issue by introducing DL-based AC methods.

Several studies employ the U-Net CNN to estimate the NAC image directly or generate the attenuation maps that deliver the AC image. In [47], Yang et al. used a Deep CNN to generate the attenuation-corrected SPECT from the NAC scan. The study involved 100 participants. The effectiveness of the proposed method was verified by voxelwise and segment-wise analyses against the reference, CT-based AC using the 17-segment myocardial model of the American Heart Association under a tenfold cross-validation procedure. Voxelwise correlations with the reference image were 97.7% ± 1.8% (slope, 0.94; R2 = 0.91), whereas the segmental errors stayed mostly within ± 10%. The generated Polar Maps were visually assessed for artefact reduction. The study showed promising results, but the performance of the proposed method was affected by the amounts of attenuation introduced between the scans and the different observed uptake patterns. In another work, Mostafapour et al. [48] analyzed the direct attenuation correction of SPECT MPI images, utilizing two DL-based algorithms, ResNet and U-Net. The dataset consisted of 99 patients, including both normal and abnormal cases. Moreover, the Chang AC approach [49] was applied for comparison against DL models. Based on the quantitative metrics and external evaluation of 19 images, the DL approaches produced images that agree with SPECT CT-AC images, whereas the Chang approach underestimated the patient’s status based on the horizontal profile. ResNet and U-Net achieved a ME of 6.99 ± 16.72 and − 4.41 ± 11.8 and an SSIM of 0.99 ± 0.04 and 0.98 ± 0.05, respectively. The Chang approach extracted the ME and SSIM of 25.52 ± 33.98 and 0.93 ± 0.09, respectively.

Mostafapour et al. [50] investigated the generated attenuation-corrected images utilizing ResNet and U-Net. This research enrolled 99 patient cases. NAC SPECT images were included as input, and CT-based attenuation-corrected images were used as reference. Nineteen cases were provided as an external validation dataset to further evaluate the models. Chang’s method [49] was compared against the deployed ResNet and U-Net approaches and was found inferior, with a ME of − 6.99 ± 16.72, against − 4.41 ± 11.8, and an SSI of 0.99 ± 0.04 and 0.98 ± 0.05, respectively. Chen et al. [51] explored the capabilities of transfer learning and utilized the state-of-the-art networks U-Net and DuRDN to generate attenuation maps from SPECT. A total of 200 SPECT/CT cases were included in this research. Regarding the results, DuRDN outperformed the prediction of μ-maps and the reconstruction of SPECT AC images. The concluded error between ground-truth and predicted μ-maps is 5.13 ± 7.02 and between ground-truth and reconstructed images is 1.11 ± 1.57%.

Chen et al. [52] compared the efficiency between direct and indirect techniques for dedicated SPECT and general purpose SPECT datasets by developing U-Net and DuRDN. In both approaches, AC was performed using CT-derived μ-maps as ground truth. More specifically, in indirect methodologies, attenuation maps (μ-maps) are generated from emission images, whereas in direct methodologies, attenuation-corrected (AC) images are predicted directly from non-attenuation (NAC) images without the need for μ-maps. Concerning general purpose SPECT, the study involved 400 participants, who underwent stress and rest examinations, where in the direct approach, both photopeak and scatter images were concatenated and inserted as input into the networks to predict the corresponding AC images directly. In indirect methodologies with general purpose SPECT, the NAC images were first concatenated. They were then applied as input to the networks to predict the intermedia μ-maps, which were then utilized for the iterative reconstructions to output the predicted AC images. The indirect strategies with DuRDN as a DL approach for dedicated SPECT with full μ-maps achieved better results with an nMSE (average normalized Mean Squared Error) of 1.2 ± 0.72%, in contrast to the 2.21 ± 1.17% yielded by past direct methodologies. Overall, for both SPECT systems, the indirect approaches demonstrate stability and efficiency in contrast to direct approaches, where the direct image-to-image transformation might not ensure constancy.

GANs enjoy remarkable success in generating the AC image directly from the NAC input. For example, Shi et al. [53] developed a 3D CNN based on a cGAN framework to estimate attenuation maps directly from emission data. Sixty-five cardiac SPECT images were included, where both photopeak and scatter were inserted. Clinical characteristics such as gender, age, height, weight, and BMI were also incorporated. The corresponding patients went on a 1-day stress-only low-dose protocol. The proposed model achieved an nMAE of 3.6%0.85% on a test set of 25 images, ensuring that the model can generate trustworthy attenuation maps consistent with CT-based maps. Liu et al. [54] explored the potential of the PRAC (Post-Reconstruction Attenuation Correction) approach combined with DL methodology to provide accurate AC images for SPECT systems. The study included 30 SPECT clinical cases in stress demonstration. The researchers developed a 3D GAN model to synthesize the attenuation map directly from the NAC SPECT image. Following this, the PRAC image was reconstructed utilizing the synthesized map and the virtual projections. For further evaluation, the PRAC image was generated based on the DL attenuation map and the CT-based attenuation map. The results were compared with scanner-generated AC images to serve as the reference ground truth. Following the post-reconstruction AC, both approaches performed consistently in contrast with scanner-generated NC images. Overall, the PRAC method with both approaches can enhance the correlation with the scanner-generated AC images compared with scanner-generated NC images. Furthermore, PRAC-CT outperformed PRAC-DL regarding scattering. In terms of metrics, the PRAC-CT extracted SSIM of 0.946 ± 0.041 compared to the PRAC-DL of 0.902 ± 0.056. However, both methodologies reduce ROI biases after attenuation correction. The authors developed a PRAC approach based on scanner-generated NC images without adding raw data from CT-less attenuation correction.

Shanbhag et al. [55] developed a conditional generative adversarial neural network model to generate simulated AC images straightly from NAC images, without including the use of CT. The dataset included 4886 patients, where short-axis NC and AC images are demonstrated for training purposes and 604 patients from two separate external sites included for testing purposes. For comparison reasons the authors gathered the results of stress TPD attained from NC, AC and DeepAC (generated of the proposed model) images. The proposed model achieved 0.79 AUC compared to NC TPD with 0.7 AUC and similar with AC TPD. With respect to normalcy rate the generated simulated images produced better results with 70.4% and 75.0% for DeepAC TPD and AC TPD accordingly, in contrast with NC TPD which extracted 54.6%. As a conclusion, the developed model enhanced the diagnostic accuracy for obstructive CAD and can function without the need of CT hardware and produced results similar to actual AC images.

Hagio et al. [56] proposed a convolutional neural network based on deep learning methodology to generate “virtual” attenuation-corrected polar maps from NAC data, without adding CT imaging scans. The study includes 11,532 cases with paired NAC and CTAC images. The authors developed a DL algorithm based on the U-Net architecture framework to predict DLAC polar maps from NAC polar maps. The produced model attained 0.827 AUC, in contrast with NAC images, which extracted 0.78 AUC. Regarding sensitivity and specificity, the produced model extracted 88% sensitivity and achieved 18.9% increased value of specificity for DLAC and 25.6% for CTAC polar maps. Conclusively the developed model generated similar attenuation-correction images with CTAC and accomplished better diagnostic accuracy with exceptional overall performance.

Similar results have been reported in other studies as well [56, 57]. Nguyen et al. [58] developed a 3D-GAN with U-Net as a generator to produce AC images. The study involved 603 patients under SPECT MPI stress and rest demonstration; however, only stress cases were included in the research. The proposed model was evaluated against CAE-based and GAN-based architectures, and the 3DU-Net-GAN network outperformed with SSIM 0.945% and NMAE 0.034. Torkaman et al. [59] proposed a 3D conditional GAN for producing AC SPECT images directly from NAC images. A total of 100 SPECT/CT images were involved. Unlike traditional methods, the suggested approach does not require intermediate attenuation maps, which depend on the generation of CT counterparts utilized as input for attenuation correction. According to the results, the proposed cGAN can generate AC images without utilizing CT data. The reference CT-based correction yielded nRMSE of 0.2258 ± 0.0777, greater than that of the cGAN (0.1410 ± 0.0768). Similar performance was reported when considering the PNSR and the SSIM values.

#### De-noising

SPECT images suffer from noise and artefacts. Metallic implants, patient motion, contrast medium, and truncation typically produce image artefacts affecting the SPECT quantification procedure [60].

The U-Net CNN is occasionally utilized for down-sampling and up-sampling the noisy input image to improve its quality. Liu et al. [61] presented a coupled U-Net modification and compared it against the iterative OSEM algorithm with three-dimensional (3D) Gaussian post-filtering for SPECT image denoising. The experiment involved 895 clinical studies. The authors used the non-prewhitening matched filter (NPWMF) to evaluate the performance of perfusion defect detection. Their method achieved a significant (8%) increase in the signal-to-noise ratio (SNRD) in the NPWMF output, and the denoised images significantly improved the detection performance of perfusion defects. In a subsequent study [62], the same framework was evaluated on 190 SPECT subjects. ROC analysis on reconstructed images with and without processing by the DL network using a set of clinical SPECT-MPI was performed. Again, compared to the Gaussian post-filtering reconstruction, the proposed DL network achieved greater de-noising, increasing the AUC from 0.80 to 0.88. Kikuchi et al. [63] explored the automatic extraction of myocardial regions from SPECT images to reduce the negative impact of extracardiac activity in the patient. Six hundred ninety-four myocardial SPECT images were included in stress and rest conditions. Various deep neural network architectures were proposed, such as 1-layer U-Net, 4-layer U-Net and 4-layer U-Net++. A multi-slice input method was also developed for the training procedure in order to improve myocardial detection performance. Reviewing the results, U-Net++ performed tremendously and decreased the effects of the extra-myocardial activity. Its structure was characterized as useful. The Dice coefficient was 0.918 at the pixel level, and there were no false positives at the slice level using U-Net++ with 9 input slices.

GANs can serve as denoising CNNs as well, aiming to estimate the low-noise counterpart, given a noisy image. There are three works employing such CNNs for this task. By developing a GAN model, Mok et al. [64] aimed to reduce the noise level in each SPECT dual respiratory and cardiac gating case produced from an XCAT phantom. The study involves 120 realistic noisy projections simulated from RAO (Right Anterior Oblique). The results revealed that with the application of GAN, the noise level gradually reduced for the DG reconstructed images, extracting NSD 0.514 and 0.215 for without and denoising, respectively. However, when the DG images formed a cardiac image, the NSD were 0.229 and 0.093 for without and with denoising. Sun et al. [65] developed a denoising method for dual gating MP-SPECT images utilizing a 3D cGAN. Twenty patients underwent stress examinations and produced 5D SPECT/CT scans. Twenty extended phantoms were also included in the simulation, where six respiratory and eight cardiac gates were demonstrated. The results have demonstrated that both simulation and real data and the cGAN approach can reduce the image noise effect and provide better image quality. The authors compared their method with post-reconstruction image improvement filters. The noise level was substantially reduced from 0.1671 ± 0.078 to 0.0520 ± 0.023 with the cGAN approach, whereas conventional filtering methods achieved a minimum of 0.0902 ± 0.051 noise level. Sun et al. [66] developed a GAN structure to decrease the noise effect in SPECT images. A total of five XCAT phantoms were included, where, for each phantom, six respiratory and eight cardiac gates were used. Overall, 48 DGs were included. Regarding the clinical dataset, one clinical patient underwent stress SPECT/CT, where the dataset was re-binned into seven respiratory and eight cardiac gates. Overall, 56 DGs were included. Based on the results, the clinical data provides superior performance to denoising along with the development of GAN, achieving NSD 0.083 and FWHM 1.489.

#### Reconstruction

Dietze et al. [67] presented a custom CNN to increase the quality of SPECT/CT images and compared the results against standard methods like Monte Carlo-based reconstruction, FBP (Filtered Back Projection), and CLINIC (Clinical Reconstruction). A total of 128 SPECT/CT scans were included in the study. The researchers proposed generating a low-quality reconstructed image utilizing FBP and then inserting the result into a deep convolutional encoder–decoder neural network to improve the image quality. The research demonstrated that FBP with the combination of CNN for image enhancement in SPECT/CT images could apply reconstruction in a minimized time with similar results to the Monte Carlo-based reconstructions, which perform slower. The mean squared error of the neural network approach in the validation set was between the Monte Carlo-based and clinical reconstruction, and the lung shunting fraction difference was lower than 2 per cent. In another study, Chrysostomou et al. [68] investigated the capabilities of DL methodology and, more specifically, CNNR (CNN Reconstruction) with encoding–decoding capabilities for SPECT image reconstruction, with the intent to retain patient radiopharmaceutical injection to minimum levels. The study includes 600,000 software phantoms for training the network. The proposed network was compared against FBP, MLEM (Maximum Likelihood Expectation Maximization) and OSEM (Ordered Subset Expectation Maximization), where CNNR achieved an SSIM of 0.938 and FBP, OSEM, and MLEM attained 0.834, 0.929, and 0.928, respectively, for low noise methodology.

In [69], Song et al. explored a post-processing technique to reconstruct 4D SPECT images utilizing a 3D residual CNN. A total of 197 clinical cases were included. The training of CNN consists of reconstructed gated images as input and their corresponding counterparts acquired from the 4D reconstruction algorithm as output, which means that the CNN will be trained to map gated reconstructed images as the result of 4D reconstruction. The proposed CNN demonstrated that it could suppress the noise level in the reconstructed myocardium and outperformed two standard methods, NLM and Gaussian post-filter. CNN achieved an NMSE of 0.033, whereas NLM and Gaussian post-filter obtained 0.04 and 0.042, respectively. In addition, the proposed CNN model decreases the MSE by 17.5% and 21.4%, in contrast with the NLM and Gaussian filters, respectively.

Xie et al. [70] explored the potential to add projection angular sampling data to improve the reconstruction process and developed a U-Net structure for this purpose. Their study used pig and physical phantom data to evaluate the DL methodology. The results were compared with the multi-angle reconstruction approach. U-Net receives one-angle images and outputs multi-angle images. According to the results, increasing angular sampling can enhance the image quality. The U-Net structure and multi-angle approach generate enhanced-quality images compared to their one-angle counterparts, and the U-Net structure was found to be outstanding in clinically used one-angle results. More importantly, U-Net outperforms the generation of images since it is not always suitable to obtain multi-angle data.

#### Other imaging improvements

Other image improvements and manipulation methods include super-resolution and reorientating cardiac SPECT images. The reorientation of SPECT images refers to an approach wherein the transaxial cardiac SPECT images are reoriented into the standard short-axis slices.

Cheng et al. [71] developed a super-resolution reconstruction network based on residual architectures to improve the resolution of SPECT images. Thirty-five sets of XCAT phantoms were used for training, and three sets of XCAT were used for validation. The study focused on 2D parallel beam reconstruction, where the input is an LR sinogram, and the output is an SR sinogram. Following the SR projection image acquisition, the SR image is reconstructed. According to the results, the proposed method achieved better PSNR and SSIM and executed superior noisy sinograms in contrast to traditional methods.

Zhang et al. [72] proposed a CNN structure to reorient SPECT images for accurate processing and analysis. The dataset includes 322 patients in the stress demonstration. Seventy-five images were utilized as external validation. The proposed CNN predicted six rigid-body transformation parameters, and then a spatial transformation network was developed to employ these parameters on the input images for generating reoriented images. Furthermore, polar maps were acquired from the produced images, and the average count values from 17 segments were calculated to estimate the quantitative accuracy of the presented method. According to the results, all images were reoriented effectively and the average count of 17 segments complied with the reference manual method.

It is worth noticing that other imaging improvements include motion correction and artefact removal. Several studies are addressing such issues in the PET modality [73]. In the current review, such studies were not found in the literature. Nevertheless, those challenges also apply to SPECT imaging, opening the horizons for future studies.

## Discussion and conclusions

### Major objectives/major findings

#### CDV detection/classification

Four major objectives have been identified in related studies concerning CDV detection. Firstly, the development of AI methods for diagnosing Coronary Artery Disease based on ICA findings. Secondly, developing frameworks that strongly agree with the human interpretation of SPECT images in identifying abnormal/ischemic/defected locations in segmented Polar Maps or identifying CAD risk based on the complete SPECT scan. Thirdly, per-vessel detection of defects and abnormalities is based on visual interpretation by human experts. Finally, the development of pipelines that exhibit superior performance to quantitative analysis, such as Total Perfusion Deficit in the tasks mentioned above.

With reference to ICA findings, most of the studies report an accuracy of 82 ± 5%. It is worth mentioning that most diagnostic tests, such as MPI, Dobutamine stress test, and ECG, exhibit sub-optimal performance. For example, in the data collected in [34], MPI yielded an accuracy of 75%, a sensitivity of 76.94%, and a specificity of 70.31%. DL approaches improved this performance by at least 10%. Ensemble models use image data and clinical attributes, thereby approaching the problem from a broader perspective. Besides, the diagnostic tests do not always reflect predisposing factors and recurrent diseases that significantly affect the risk of suffering from cardiac diseases. Diagnostic tests may overestimate or underestimate the situation. AI manages to integrate complex factors and image processing to predict the outcome.

Reporting whether the study examines the agreement rating with the visual interpretation by the experts or the accuracy in detecting CAD based on reliable labelling of the dataset is essential. Only 28.6% per cent of the studies train their networks to predict the presence of CAD, whilst other studies compare their results with the visual interpretation performed by the experts, thereby measuring the agreement between AI and humans. In the case of CAD, SPECT imaging performs sub-optimally [35], yielding false positives and negatives. SPECT imaging exhibits an AUC of 0.83 in [74], sensitivity and specificity of 0.84 and 0.69 in [75], and sensitivity and specificity of 0.83 and 0.41. In such cases, AI methods are not expected to perform significantly better due to the nature of the images. More specifically, significant features that are not visible to the human eye seldom appear, so there is a limitation from the outset regarding how accurate an artificial intelligence model can be in diagnosing diseases from such images.

On the contrary, studies that address the efficiency of their models based on human experts’ diagnostic yield are expected to exhibit much better results. Many studies have used nuclear medicine experts’ diagnostic yield as their gold standard. They aimed to compare AI methods against the human eye and interpret the results. Such studies reported a remarkable agreement rate between the AI prediction and the experts' diagnosis. For example, Selcan Kaplan Berkaya et al. [32] achieved a 92% agreement rating. Papandrianos et al. [19] achieved a rating of 93.48 ± 2.81%. The overall agreement rating ranged from 86 to 93%.

DL methods are found to be superior to conventional quantitative assessment. In all the reported studies, DL models improved the diagnostic accuracy by approximately 10%. Besides, DL methods yielded better AUC scores. For example, in [24], the AUC score for disease prediction by DL was higher than for TPD (per patient: 0.80 vs. 0.78; per vessel: 0.76 vs. 0.73). With the DL threshold set to the same specificity as TPD, per-patient sensitivity improved from 79.8% (TPD) to 82.3%, and per-vessel sensitivity improved from 64.4% (TPD) to 69.8%.

Finally, excellent agreement ratings with the human interpretation were reported in the localization performed on a fine 17-segment division of the polar maps. For example, in [31], graph convolutional neural networks achieve an agreement of 83.1% with the human observer.

#### Image quality improvement

Three major objectives were examined within the category of image quality improvement. Firstly, some studies focused on SPECT image de-noising and, more specifically, artefact removal. Secondly, several studies proposed DL-based methods to estimate the AC SPECT image using the uncorrected counterpart. Thirdly, some studies proposed DL methods to estimate standard dose and standard time SPECT scan images using their low-dose and fast-scan counterparts.

Concerning image de-noising, the proposed CNNs outperformed conventional image denoising algorithms and methods, such as the well-established three-dimensional (3D) Gaussian post-filtering. The classic U-Net was employed in most of the studies with specific modifications.

The direct attenuation correction achieved by DL methods, such as the U-Net and GAN topologies, reported metric results that are similar or superior to the conventional attenuation correction methods. Their results are remarkable despite the small-scale datasets used to train such networks in the current literature. However, visual inspection of the corrected images is not present in most cases. Image similarity metrics, though undeniably reliable, need to be accompanied by visual comparisons performed by experienced medical experts. CNNs and GANs have been designed and successfully deployed for denoising and reconstruction purposes with similar results.

The estimation of the full-count SPECT scan from the low-count SPECT has been the focal point of a few studies. CAE, GANs, and ResNet are the most famous strategies in the literature. They are reported to accurately estimate the full-count SPECT image given the 1/8 and 1/16 standard clinical dose image or the half acquisition time output. Though managing to exhibit similar or fewer errors (RMSE) than the conventional methods, the trade-off between radiation exposure reduction and clinical information loss has not been investigated yet.

**Fig. 7:**
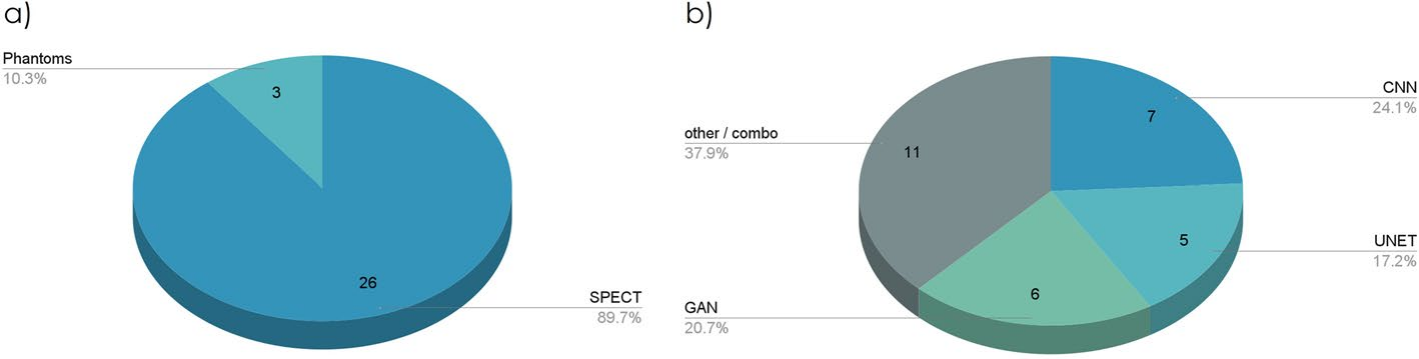
Proportion of image quality improvement studies in terms of: **a** learning type (CNN, GANs, UNET or combinations of them), **b** data source (SPECT images and phantoms)

In terms of the employed algorithm, combination of U-Net, GAN, and CNN is found in most of the reported studies (37.9%), as seen in Fig. 7.

### Clinical factors can improve the diagnostic accuracy

Integration of clinical factors would aid in the explainability issue and can improve the diagnostic accuracy of the models. Some studies supply demographic characteristics to their models, such as the gender and sex of the subject. The importance of the demographic factors in the final prediction is not examined further. In the study of Apostolopoulos et al. [35], the authors used 23 clinical attributes and the DL model’s prediction on the image to build a Random Forest classifier. There was a 14% improvement in accuracy when the clinical attributes were embedded into the model. However, the importance of each attribute was not further examined.

Future research could involve both images and clinical information. Studies that perform clinical information feature analysis and selection, whilst employing explainable techniques such as the Grad-CAM algorithm for the involved images, are expected to provide better results and be more explainable. Before the era of DL, cardiovascular disease detection relied on clinical attributes in most studies. Such studies offer great insight into the relationship between the clinical attributes and could advise how such attributes can be analyzed and embedded into the model.

### Hand-crafted and pre-trained CNNs

**Fig. 8:**
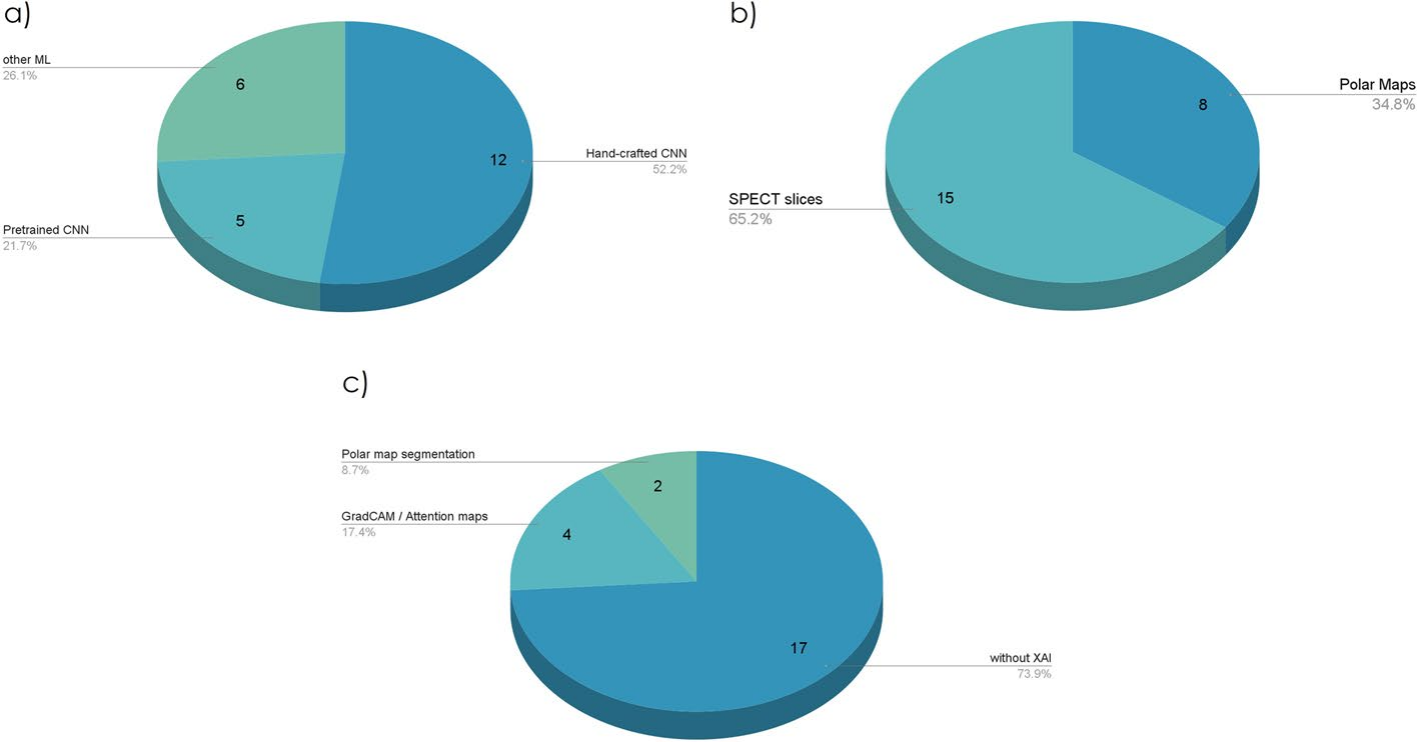
Proportion of diagnosis classification studies in terms of: **a** learning type employing pretrained networks and hand-crafted networks, **b** data source employing SPECT images and Polar Maps, and **c** explainability employing GradCAM, attention maps, polar map segmentation techniques or without any XAI tool

Most methods use handcrafted CNNs (52.2%), as seen in Fig. 8. On the contrary, few studies employ state-of-the-art pretrained networks, such as VGG and ResNet. Handcrafted CNNs seem preferable, perhaps due to their minimum complexity. Besides, the nature of the images does not pose additional challenges that would require millions of trainable parameters and sufficient depth. CNNs designed for cardiovascular disease diagnosis from SPECT images are not expected to discover novel image biomarkers or complex patterns inside the image. Moreover, most studies involve small-scale datasets that are inherently unsuitable for training large and deep networks. Despite those facts, it is observed that both strategies yield equivalent results. This is because studies which employ pretrained networks usually freeze some of their layers, thereby reducing the trainable parameters and retaining their depth. In essence, they adjust those networks to the needs of the particular datasets.

Effective Medical Decision Support Frameworks require lightweight models to facilitate their prediction in real-time during the daily routine. Hence, it would be interesting to report the training and testing times of the models. Training and testing times depend heavily on the number of trainable parameters each network introduces, the general network structure and depth, and the input data format (2D, 3D, etc.). In the study of Berkaya et al. [32], the authors observed that the proposed VGG-16 network required 0.43 s to classify a new image, whereas the rule-based approach required 4 s. From a technical point of view, it is a limitation that most of the studies do not report the training and testing times of the developed networks.

### Robustness to SPECT scanner variation

SPECT scanners differ from each other, introducing extra challenges. DL may identify variabilities between sites, camera types, and tracers as important features. Using the polar map format resolves such variabilities. However, that is not the case in many studies. Hence, the suggested pipelines may perform differently in unseen data than other devices. Some works in the literature seek to examine this property of the developed models [34, 35]. The developed frameworks show some robustness to image acquisition device variation, though this issue is not rigorously examined in current research. Multicenter studies give the overall picture more comprehensively since the control of the models is done using images from different scanners. In addition, studies that utilize Polar Maps might circumvent these issues because they do not interact with the raw SPECT image directly. The review identified some studies based on Polar Maps (34.8%), as observed in Fig. 8.

Most studies that achieve image quality improvement (attenuation correction, de-noising, etc.) do not evaluate their methods on different scanners. This can be considered a limitation of the current research. On the contrary, studies on disease classification often tested their networks for robustness to SPECT scanner variation.

### The explainability issue

Deep Learning methods face the explainability issue [76]. Since medical decisions are undeniably based on strict and specific criteria and guidelines, the inability of DL to present how and why it performed the desired task is a major obstacle to becoming an established pipeline in everyday practice. At present, DL is found to be excellent in extracting features, detecting artefacts, tissues, tumours, segmenting parts of the body and organs, and generating synthetic data, but severely weak in revealing crucial cause and effect relationships that would aid the current medical research towards finding new image biomarkers, at least at present. The act of DL as a black box makes the medical community reluctant to adopt DL in assisting with everyday challenges. There is an increasing demand for transparency and interpretability of the new methods.

The literature review identified many studies that use explainability techniques. It is worth noting that most studies do not use any explainable methods (Fig. 7). Three studies used the Grad-CAM algorithm to visualize important regions of the image based on the model’s prediction. In the study of Chen et al. [30], the visualization pointed out areas with myocardial defects. However, a visual assessment of the entire test set is not provided.

Otaki et al. [22] used attention maps for each segment of the standard 17-segment American Heart Association model to generate the segmental CAD probability map. The CAD probability map marks the degree to which the segments contribute to the model’s prediction. Similar approaches were followed in [31, 37]. Although the techniques mentioned above do not reveal the internal decision-making procedure of the model, they help address the model’s performance in detecting the right areas of the image.

Though explainability is highly desirable in image classification tasks, improving the image quality does not require rigorous explainability tools. However, the black-box nature of DL methods still raises concerns, especially when compared to conventional denoising methods, which are highly transparent.

## Concluding remarks

The review addressed major objectives and findings in cardiac SPECT imaging using the recent advances in DL methods. The research identified 52 studies. Five major application categories have been distinguished: disease classification, image de-noising, attenuation correction, full-count SPECT estimation, and reconstruction. It can be concluded that DL methods exhibit strong agreement ratings with human experts, are superior to the traditional quantitative assessment, and can effectively diagnose cardiovascular disease in a non-invasive manner with an accuracy of 82 ± 5%. Besides, DL methods are remarkably effective in image quality improvement tasks, obtaining high precision in estimating the AC SPECT image and the full-count SPECT image. Image reconstruction and de-noising are also investigated using DL methods and yield promising results. Limitations—future research directions include (1) the small-sized datasets that are currently available and the sparse deployment of advanced data augmentation techniques; (2) the occasional use of clinical attributes to enhance the effectiveness of the DL models; (3) the lack of many multi-centre studies to verify the robustness of the developed frameworks; and (4) the establishment of explainability tools and algorithms to evaluate the effectiveness and enhance the reliability of the proposed methods.

## Data Availability

Not applicable.

## Abbreviations

AB: Adaptive boosting
AC: Attenuation correction
AI: Artificial intelligence
ANN: Artificial neural network
ARE: Absolute relative error
CAD: Coronary artery disease
CLINIC: Clinical reconstruction
CNN: Convolutional neural network
DL: Deep learning
FBP: Filtered back-propagation
GAN: Generative adversarial network
GB: Gradient boosting
Grad-CAM: Gradient-weighted class activation mapping
MAGE: Major adverse cardiac events
ME: Mean error
ML: Machine learning
MLEM: Maximum likelihood expectation maximization
NAC: Non attenuation correction
nME: Normalized mean error
NPWMF: Non-prewhitening matched filter
nRMSE: Normalized root-mean-squared error
NSD: Noise level measured as the normalized standard deviation
OSEM: Ordered-subsets expectation–maximization
PET: Positron emission tomography
PRAC: Post-reconstruction attenuation correction
PSNR: Peak signal-to-noise ratio
RAO: Right anterior oblique
RF: Random forest
RMSE: Root-mean-squared error
ROC: Receiver operating characteristic
SOTA: State of the art
SPECT: Single-photon emission computerized tomography
SSIM: Structural similarity index measure
ST-NLM: Spatiotemporal non-local means
SVM: Support vector machine
TPD: Total perfusion deficit
XGB: Extreme gradient boosting
